# Non-additive dosage-dependent effects of *TaGS3* gene editing on grain size and weight in wheat

**DOI:** 10.1007/s00122-025-04827-w

**Published:** 2025-01-29

**Authors:** Wei Wang, Qianli Pan, Bin Tian, Zitong Yu, Dwight Davidson, Guihua Bai, Alina Akhunova, Harold Trick, Eduard Akhunov

**Affiliations:** 1 Wheat Genetics Resource Center, Kansas State University, Manhattan, KS USA; 2 Nanjing Agricultural University, Nanjing, China; 3 USDA-ARS Hard Winter Wheat Genetics Research Unit, Manhattan, KS USA; 4 Integrated Genomics Facility, Kansas State University, Manhattan, KS USA

## Abstract

**Key message:**

Loss-of-function mutations induced by CRISPR-Cas9 in the *TaGS3* gene homoeologs show non-additive dosage-dependent effects on grain size and weight and have potential utility for increasing grain yield in wheat.

**Abstract:**

The grain size in cereals is one of the component traits contributing to yield. Previous studies showed that loss-of-function (LOF) mutations in *GS3*, encoding G*γ* subunit of the multimeric G protein complex, increase grain size and weight in rice. While an association between allelic variation in the *GS3* homologs of wheat and grain weight/size has been detected previously, the effects of LOF alleles at *TaGS3* on these traits remain unknown. We used genome editing to create *TaGS3* mutant lines with varying LOF homeo-allele dosages. Contrary to the results obtained in rice, editing all three *TaGS3* homoeologous copies resulted in a significant decrease in grain length (4.4%), width (3.4%), grain area (7.3%) and weight (7.5%), without affecting the number of grains per spike. Compared to the wild type, the highest increase in grain weight (up to 9.6%) and area (up to 5.0%) was observed in homozygous mutants with one or two genomes carrying LOF homeo-alleles, suggesting non-additive suppressive effects of *TaGS3* on grain size and weight in wheat. Our results suggest that the regulatory effects of *GS3* homologs in wheat and rice have diverged. The newly developed LOF homeo-alleles of *TaGS3* expand the set of CRISPR-Cas9-induced variants of yield component genes that have potential to increase grain weight in wheat.

**Supplementary Information:**

The online version contains supplementary material available at 10.1007/s00122-025-04827-w.

## Introduction

Genetic analyses of yield component traits, mostly performed in rice, identified a number of genes involved in pathways controlling grain size and weight in cereal crops (Li and Li 2016; Li and Yang 2017). Just few examples of many characterized grain size/weight QTL loci identified in rice are *GW2* (Song et al. 2007), *GW8* (Wang et al. 2012), *GW7* (Wang et al. 2015), *GS5* (Li et al. 2011), *GS2* (Hu et al. 2015), *GSE5* (Geng et al. 2017), *OsCKX2* (Ashikari et al. 2005), *DEP1* (Huang et al. 2009) and *GS3* (Fan et al. 2006). Identification of these genes along with the development of comparative genomic resources accelerated genetic dissection of yield component traits in other crops, including wheat. The analysis of natural allelic diversity and/or mutagenesis performed in wheat showed that many rice homologs of the grain size and weight genes are linked with variation in the same traits (Li and Yang 2017; Wang et al. 2019). For example, both gene editing and mutagenesis showed that the loss-of-function mutations in *TaGW2* gene encoding RING-type protein with E3 ubiquitin ligase increase grain size and weight (Simmonds et al. 2016; Wang et al. 2018b). The editing of *TaGW7* gene encoding TONNEAU1-recruiting motif protein demonstrated its involvement in regulation of grain shape and weight in wheat (Wang et al. 2019). Likewise, natural variation in the wheat orthologs of rice yield component genes, *TaCKX2* (Zhang et al. 2012), *TaGL3* (Yang et al. 2019a) and *TaGS3* (Yang et al. 2019b), was associated with variation in grain weight traits in wheat populations.

One of the well-investigated pathway contributing to grain size variation is mediated by G protein complex composed of G*α*, G*β* and G*γ* subunits (Thung et al. 2012; Urano and Jones 2014). In rice, *GS3* gene encoding the atypical G*γ* subunit was shown to act as negative regulator of grain size and weight (Fan et al. 2006). It was shown that GS3 competes for interaction with the G*β* subunit with two other atypical G*γ* subunits, DEP1 and GGC2, that act as positive regulators of grain size and weight (Sun et al. 2018). The N-terminal organ size regulation (OSR) domain in *GS3* was shown to be critical for negative regulatory effects (Mao et al. 2010). Loss-of-function mutations in *GS3* or mutations leading to the truncation of the OSR domain were shown to remove the repressive effect of *GS3*, resulting in longer grain and increase in grain weight (Sun et al. 2018). The strongest positive impact on grain length in rice was obtained in lines with a knockout allele of *GS3* and the overexpressed *DEP1* and *GGC2* genes (Sun et al. 2018)*.* The allelic variation in *GS3* was shown to contribute to variation in grain size and weight in natural populations and played important role in increasing rice productivity (Mao et al. 2010).

In wheat, significant associations were found between natural variation in the *TaGS3-4A* and *TaGS3-7A* homoeologs and grain weight and length (Yang et al. 2019b; Zhang et al. 2020), with different splicing variants of the gene having distinct effects on these traits (Ren et al. 2021). Interestingly, overexpression of five splicing variants of *TaGS3* in wheat showed that one of the gene isoforms can have positive effects on grain size and weight (Ren et al. 2021). It was suggested that alternative splicing resulting in truncation of the OSR domain in this *TaGS3* isoform reduces its affinity to G*β* domain, reducing its negative impact on grain size and weight (Ren et al. 2021). These studies suggest that the *TaGS3* gene, like its homolog in rice (Fan et al. 2006), should also act as a negative regulator of pathways controlling grain size and weight. Therefore, one might expect that the complete knockout of *TaGS3* in wheat should have strong positive effect on grain size and weight.

Here, we used CRISPR-Cas9-based gene editing system to induce LOF mutations in the homoeologs of the *TaGS3* gene. The effects of gene editing on grain morphometric traits, grain weight and grain number per head were investigated in the advanced generation populations derived from independent transformation events. Contrary to expectations based on prior functional studies of *GS3* in rice and *TaGS3* in wheat, the triple homozygous knockout mutants of *TaGS3* with non-functional alleles in all three wheat genomes showed significant decrease in grain length, width, grain area and weight, without discernable changes in the number of grains per spike. The highest increase in grain weight and area was obtained in the lines carrying the intermediate number of LOF alleles, suggesting that suppressive effects of *TaGS3* on grain traits in wheat are non-additive. These results suggest that *TaGS3* in wheat and its rice homolog *GS3* are not functionally equivalent and that the intermediate levels of *TaGS3* expression are likely necessary for the optimal expression of the grain size and weight traits in wheat. Our study also identified single and double mutants of the *TaGS3* homoeologs that have positive impact on grain size and weight that could be used for improving yield potential in wheat.

## Materials and methods

### Design and validation of the CRISPR-Cas9 sgRNAs targeting the TaGS3 gene

The coding sequence of the rice *GS3* gene (Fan et al. 2006) was used to perform BLASTN search against the wheat reference genome RefSeq v2.0 (The International Wheat Genome Sequencing Consortium (IWGSC) 2018). The orthologous genes identified using the sequence similarity threshold above 80% and alignment spanning more than 80% of the *GS3* gene length were located on chromosomes 7A, 4A and 7D syntenic to rice chromosome 3. The annotated sequences of the wheat orthologs, 7A (TraesCS7A02G017700), 4A (TraesCS4A02G474000 TraesCS4A02G474000) and 7D (TraesCS7D02G015000), were downloaded from Ensembl Plants and henceforth will be referred to as *TaGS3-7A*, *TaGS3-4A* and *TaGS3-7D*, respectively. The protein sequences of genes were aligned using MUSCLE (Edgar 2004), and the boundaries of functional domains were identified by aligning the wheat sequences with their rice ortholog.

The CRISPR-Cas9 targets on *TaGS3* gene were designed as described previously (Wang et al. 2018b, 2019). The coding regions of *TaGS3* homoeologs were aligned to identified conserved sequences, which were analyzed using sgRNAscorer 1.0 (http://crispr.med.harvard.edu/sgRNAScorer). The top ranked targets were compared using BLASTN against the wheat genome RefSeq v2.0 (The International Wheat Genome Sequencing Consortium (IWGSC) 2018) to select targets with low potential for off-target editing. A total of four gRNAs were designed to target the region near the CDS start in the first exon. The sequence of each gRNA was synthesized as two complementary oligos with 4 nucleotides as overhangs at both termini (Supplementary Table 1). The oligos were annealed and subcloned into CRISPR-Cas9 plasmid pBUN421 as described (Wang et al. 2018a). The genome editing efficiencies of the designed gRNAs were estimated by transiently expressing the constructs in the wheat protoplasts followed by the amplification and next-generation sequencing (NGS) of target flanking sequences (Wang et al. 2019). The primers used for sequence-based genotyping are included in Supplementary Table 1. The Illumina reads were mapped to the genomic sequences of *TaGS3-7A*, *TaGS3-4A* and *TaGS3-7D* extracted from the Chinese Spring RefSeq v.2.1 using BWA-MEM (Li and Durbin 2009). Due to sequence divergence, individual reads from different genomic copies of *TaGS3* could be mapped to the respective genomes allowing for detection of mutations in each homoeologous copy of the gene. The types (deletion, insertion, base change) and frequency of editing events were inferred from the CIGAR codes of individual Illumina reads in the alignment files. The proportion of reads with mutations at the target sites was used for genotype calling, where sites with the proportion of mapped mutated reads < 0.2 or > 0.8 were called as homozygous and sites with the proportion of mapped mutated reads ≥ 0.2 and ≤ 0.8 were called as heterozygous.

### Development of plant material

The constructs targeting two target sites TaGS3T6 and TaGS3T8 were mixed in equimolar amounts with the pAHC20 construct carrying *bar* gene and ballistically transformed into wheat embryos of cultivar Bobwhite. The T_0_ transgenic plants were regenerated as described in our previous study (Wang et al. 2019). Three primer pairs spanning the CRISPR-Cas9 constructs were used to screen for the Cas9-positive plants by PCR (Supplementary Table 1). The editing events at the sgRNA target sites were detected by Illumina sequencing (MiSeq platform, 2 × 150 bp run) of the pooled bar-coded PCR amplicons (Wang et al. 2018a), as described above.

**Fig. 1:**
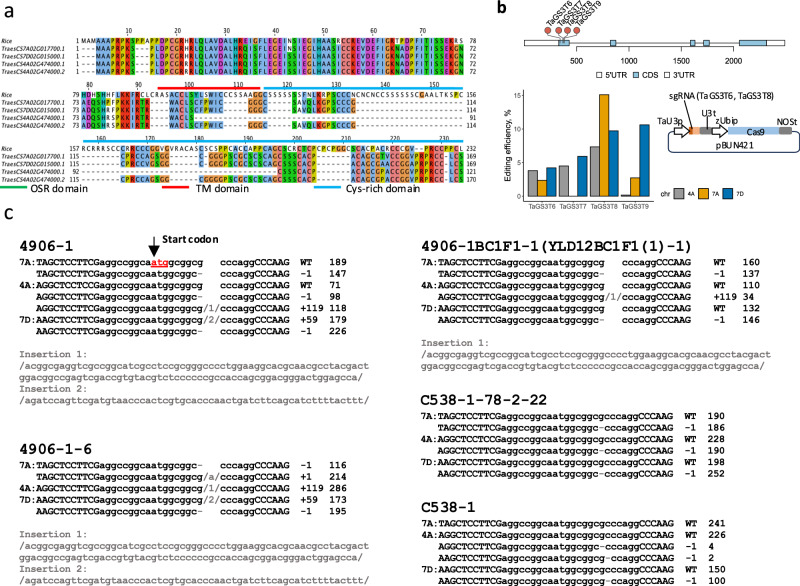
CRISPR-Cas9 editing of the *GS3* homologs in wheat. **a** Protein sequence alignment of rice and wheat GS3 homologs. The boundaries of functional domains [organ size regulation (OSR), transmembrane (TM) and cysteine-rich (Cys-rich)] are shown relative to rice GS3 protein sequence (Fan et al. 2006). **b** Distribution of four gRNA target sites in the 1st exon of the *TaGS3* gene (*TaGS3-7A* homoeolog is used as an example) conserved in all three homoeologous copies. Gene editing efficiency was assessed in the wheat protoplasts. The proportion of Illumina reads with editing events at the target sites was calculated for each *TaGS3* homoeolog by sequencing target sites using DNA extracted from the wheat protoplasts. The values were normalized by the protoplast transformation efficiency assessed using a plasmid expressing the YFP fluorescent protein. Two gene editing constructs in the background of pBUN421 plasmid targeting TaGS3T6 and TaGS3T8 sites were pooled in equimolar proportions and used for biolistic transformation of wheat cultivar ‘Bobwhite.’ **c** Genotypes of Illumina sequence reads in lines 4906-1, 4906-1-6, 4906-1-6 (BC_1_F_1_), C538-1 and C538-1-78-2-22 mapped to the homoeologous copies of the *TaGS3* gene from chromosomes 7A, 4A and 7D in RefSeq v2.1. The number of wild-type (WT) and mutated reads in the alignment is shown on the right side of the alignment. ‘− 1,’ ‘ + 1,’ ‘ + 119’ and ‘ + 59’ correspond to the number of bases deleted or inserted in the edited reads. The deleted bases are shown in the alignment as gaps (‘−’). The inserted sequences are shown below the alignments. The protospacer sequence and protospacer adjacent motifs are shown in lowercase

The lines derived from two T_0_ transgenic plants 4906-1 and C538-1, both showing the evidence of *TaGS3* gene editing, have been selected to develop two populations for evaluating the effects editing on grain morphometric traits and weight (for details see Results). The 4906-1-6 line carried edits in all three genomes (genotype *aabbdd*), whereas C538-1 was heterozygous for edits in the A and B genomes of wheat and carried no mutations in the D genome (genotype *AaBaDD*) (Fig. 1). Plant regeneration is accompanied by epigenetic modifications (Liu et al. 2023). To reduce the potential impact of epigenetic modification on phenotypic traits, line 4906-1-6 was crossed with the wild-type cv. Bobwhite to produce F_1_ hybrids, which were further backcrossed to wild-type cv. Bobwhite to produce BC_1_F_1_ plants. The self-pollinated progeny of the Cas9-negative BC_1_F_1_ plants was genotyped by the Illumina sequencing of pooled and bar-coded amplicons generated for each plant using primers flanking the CRISPR-Cas9 target sites (Supplementary Table 1).

The C538-1 line was heterozygous for the *TaGS3* LOF mutations on chromosomes 7A and 4A (genotype *AaBbDD*) (Fig. 1). The *Cas9*-negative C538-1-78-2-22 line heterozygous at all three homoeologous *TaGS3* loci was identified in the T_3_ generation of C538-1. This line was used to develop a T_5_ population including 251 lines segregating for the *TaGS3* LOF alleles in all three wheat genomes creating dosage variation of functional alleles ranging from 0 to 6.

### Plant growth conditions

The edited plants were grown in greenhouse under 16-h light/8-h dark and the temperature set at 24 °C in the day and 21 °C in the night. The T_1_ generation plants were grown in the 0.2 L square pots filled with SunGro soil (Sun Gro Horticulture, Agawam, MA, USA). All other plants were grown in 1 L square pots filled on 3/4 with soil mix having the volume ratio of soil:peatmoss:perlite:CaSO_4_ = 20:20:10:1 and 1/4 with SunGro soil mix (Sun Gro Horticulture, Agawam, MA, USA). The pots were arranged according to the complete randomized design.

### Collection of grain dimension, grain number per spike and TGW (thousand grain weight) data

The grain size (grain width, length and area), the number of grains per spike and TGW for the *TaGS3* mutants were collected using a MARVIN seed analyzer (GTA Sensorik GmbH, Germany), as described previously (Wang et al. 2019). The seeds from the three tallest spikes of each plant were used to calculate the mean values per plant.

### Gene expression analyses

For assessing the relative expression of the *TaGS3* gene isoforms, we have used RT-PCR primers developed by Ren et al. (2021). The total RNA was extracted in five biological replicates from the spikes of wild-type cv. ‘Bobwhite’ and seven *TaGS3* mutants with the following genotypes: *aabbdd*, *aaBBDD*, *AAbbDD*, *AABBdd*, *aabbDD*, *aaBBdd* and *AAbbdd*. The levels of each isoform expression were compared between the wild-type cv. ‘Bobwhite’ and each *TaGS3* mutant using the 2^−∆∆Ct^ method. The levels of *actin* gene expression were used as reference. The two-tailed *t*-test followed by the Benjamini–Hochberg (BH) procedure for multi-testing correction were used to assess the significance of expression differences.

The expression of the *TaGS3* gene homoeologs was performed using RNA-seq data available for spikes cultivar ‘Bobwhite.’ The RNA-seq data from three biological replicates were used to estimate the expression abundance of transcripts (read counts) using program kallisto (Bray et al. 2016). The reference transcripts for analyses were obtained from the RefSeq v.2.0 genome annotation. The analysis of read count data was conducted using the R package ‘DESeq2’ version 1.39.8 (Anders and Huber 2010). The read count data were transformed using the ‘*vst*’ function of DESeq2, removing the dependence of the expression variance on the mean.

### Statistical analysis of data

The distributions phenotypic data were visualized using the boxplot function from ggplot2 R package (version 3.4.4). The outliers in data were removed using the Rosner's test for outliers implemented in R package EnvStats (version 2.3.1). One-way ANOVA was applied to compare the significance of genotype effects. The Student’s *t*-test was applied to assess the significance of difference between the reference groups of edited lines (the group with the lowest number of edited *TaGS3* copies in a population) and groups of lines with the higher number of the edited *TaGS3* allele copies.

## Results

### CRISPR-Cas9 editing of TaGS3 in wheat

In the bread wheat genome, three copies of a gene showing highest similarity to *GS3* from rice are located on chromosomes 7A (TraesCS7A02G017700.1), 4A (TraesCS4A02G474000.1 and TraesCS4A02G474000.2) and 7D (TraesCS7D02G015000.1). The length of the four *TaGS3* isoforms expressed from these three homoeologs (170, 169, 121 and 169 amino acids) is shorter than that of rice *GS3* (232 amino acids) due to differences in the length of the cysteine-rich tails (Fig. 1a). While the previous study performed using the wheat cultivar ‘Kenong 9204’ showed that the levels of 7A homoeolog expression are lower than that of the 4A and 7D homoeologs (Zhang et al. 2020), we did not observe such differences in the RNA samples from the spikes of cultivar ‘Bobwhite’ used in our study (Supplementary Fig. 1b). The cysteine-rich domain was previously shown to play a role in the rate regulation of protein degradation, with longer cysteine-rich domain resulting in faster degradation of *GS3* in rice and reduced repressive effect on grain size (Sun et al. 2018). However, the effect of tail length variation on grain size is substantially smaller than the effect of the OSR domain itself, which plays major role in interaction with the G*β* (Mao et al. 2010).

To maximize the functional effect of mutations, we designed four gRNAs targeting the conserved OSR domain coding regions in the first exon of the *TaGS3* homoeologs (Fig. 1b, Supplementary Table 1). The gRNAs have been subcloned into plasmid pBUN421 and their editing efficiency was evaluated by transiently expressing constructs in the wheat protoplasts. The editing efficiency assessed by next-generation sequencing of PCR products including the gRNA target sites (Wang et al. 2021) ranged from 0 to 15.1% (Fig. 1b). Based on the efficiency of editing, we selected sgRNAs targeting the GS3T6 and GS3T8 sites for biolistic transformation (Fig. 1b). A total 67 independent transgenic plants in cultivar ‘Bobwhite’ carrying the Cas9-gRNA constructs have been regenerated. By sequencing the gRNA target regions in 22 transgenic lines, we have identified two lines, 4906-1 and C538-1, whose progeny carried heritable mutations, indicative of ~ 9.1% (2/22) editing efficiency, which is lower than 15% estimated using the wheat protoplasts. These two lines have been used for developing populations to evaluate the effects of gene editing in *TaGS3* on yield component traits. In the T_0_ line 4906-1, *TaGS3* carries heterozygous mutations on 7A, chimeric mutations on 4A and two distinct allelic mutations on 7D (Fig. 1c). In the T_0_ line C538-1, *TaGS3* has wild-type allele on 7A, wild-type allele on 4A with few reads with chimeric mutations, heterozygous mutations on 7D (Fig. 1c).

### The effects of TaGS3 gene editing on yield component traits in the BC_1_F_2_ and BC_1_F_3_ populations

**Fig. 2:**
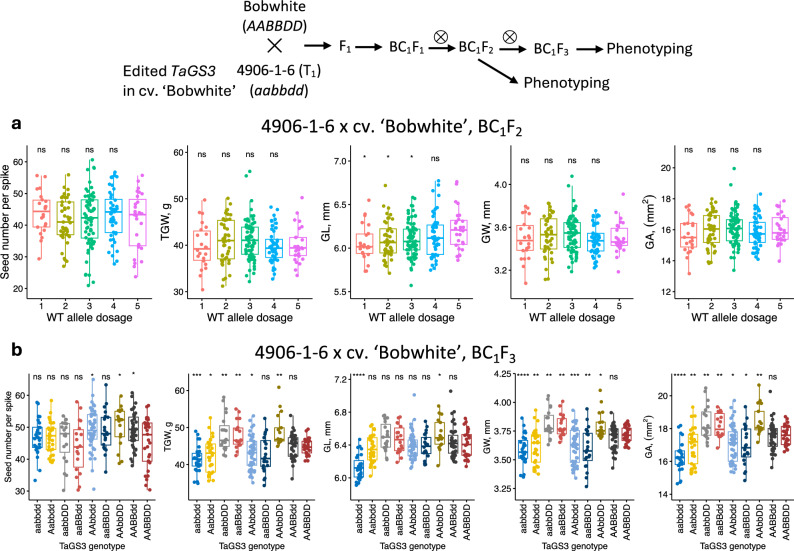
Effects of *TaGS3* gene editing on yield component traits assessed in the BC_1_F_2_ and BC_1_F_3_ populations. **a** The BC_1_F_2_ population was created by crossing 4906-1-6 line with wild-type cultivar Bobwhite. The phenotypic measurements were taken using lines grouped based on the total number of functional wild-type (WT) *TaGS3* alleles at three loci located on chromosomes 7A, 4A and 7D. **b** The phenotypic measurements were taken using the lines grouped based on their genotypes at three *TaGS3* homoeologous loci. The upper- and lowercase letters correspond to genotypes of the functional (A, B or D) and edited (a, b or d) *TaGS3* alleles, respectively. The graphs show relationships between grain number, morphometric traits and weight and the number of functional wild-type *TaGS3* alleles. The significance testing was performed by applying *t*-test to compare each genotype group with a reference group that includes lines with the highest number of functional wild-type alleles in each population. Significance levels: **** − ≤ 0.0001, *** − ≤ 0.001, ** − ≤ 0.01, * − ≤ 0.05, ns − > 0.05

To reduce the possible effects of epigenetic changes associated with the regeneration of transgenic lines on phenotypic evaluation and to fix *TaGS3* homeo-loci on chromosomes 7A and 7D for only one of the edited variants, the transgenic line 4906-1-6 (Fig. 1c), which carried the LOF alleles at all three *TaGS3* homeo-loci, was backcrossed with wild-type cultivar ‘Bobwhite’ (Fig. 2). The BC_1_F_1_ plants negative for the Cas9 construct were used for developing the BC_1_F_2_ and BC_1_F_3_ populations. In the BC_1_F_2_ population, lines derived from this cross segregated for the total number of functional *TaGS3* alleles, which allowed us to investigate the effect of allele dosage on yield component traits. The BC_1_F_2_ population was phenotyped for thousand grain weight (TGW), grain number per spike (GN), grain length (GL), grain width (GW) and grain area (GA) traits (Fig. 2). Due to small number of lines in some of the genotype classes (Supplementary Table 2), lines were further grouped based on the number of non-edited wild-type alleles and the mean phenotypic values were compared between the groups. Due to the insufficient number of homozygous BC_1_F_2_ lines carrying wild-type (*AABBDD* genotype) or mutated (*aabbdd* genotype) alleles in all three *TaGS3* homoeologs, these genotypes were not included into this analysis; the effects of these genotypes on traits were investigated in the BC_1_F_3_ population (Fig. 2b). In this population, the increase in the number of *TaGS3* LOF alleles was associated with the reduction in grain length (Fig. 2a). This trend is opposite to the effects expected from the *TaGS3* gene knockout if this gene is a negative regulator of grain length. The lines that carried five LOF homeo-alleles of *TaGS3* showed 2.5% reduction (t-test *p*-value = 0.02) in GL compared to lines with only one LOF allele of *TaGS3*. No significant effects of *TaGS3* editing were detected for other grain morphometric traits (Fig. 2a). In lines that carried intermediate number of wild-type alleles, we have also observed some increase in the TGW compared to lines that carried one or five wild-type alleles (Fig. 2a).

To further investigate the phenotypic effects of mutations in the specific homeo-alleles of *TaGS3*, we have developed BC_1_F_3_ population using lines from the BC_1_F_2_ population that carried only wild-type alleles (genotype *AABBDD*) or knockout mutations at one (genotypes *aaBBDD*, *AAbbDD* or *AABBdd*), two (genotypes *aabbDD*, *AAbbdd* or *aaBBdd*) or three (*aabbdd*) *TaGS3* loci (Table 1, Fig. 2b, Supplementary Table 3). In the BC_1_F_3_ population, we have also observed reduction in GL with an increase in the number of edited gene copies. While single- or double-gene mutants did not show significant differences in GL compared to wild-type genotype, the triple mutants showed 4.4% reduction in GL (t-test *p*-value = 1.7 × 10^−8^), confirming observations made in the BC_1_F_2_ population.

**Table 1:** Effects of CRISPR-Cas9-induced mutations in the *TaGS3* gene on grain weight, grain morphometric parameters and grain number per head in BC_1_F_3_ population derived from the 4906-1-6 × cv. Bobwhite cross

Genotype	*N*	GNH	TGW (g)	GA (mm^2^)	GW (mm)	GL (mm)
AABBDD	29	45.37 ± 7.72	44.83 ± 2.18	17.58 ± 0.61	3.71 ± 0.07	6.41 ± 0.17
aabbdd	27	46.77 ± 5.62	41.46 ± 3.63***	16.3 ± 0.92****	3.59 ± 0.13****	6.13 ± 0.15****
aaBBDD	20	48.9 ± 6.37	42.66 ± 4.96	16.97 ± 1.21*	3.6 ± 0.1	6.39 ± 0.16
AAbbDD	17	50.25 ± 6.17*	49.13 ± 4.4**	18.46 ± 0.91**	3.8 ± 0.12	6.53 ± 0.19*
AABBdd	39	49.06 ± 5.57*	45.04 ± 3.33	17.55 ± 0.87	3.69 ± 0.11	6.44 ± 0.19
aabbDD	19	45.93 ± 6.8	48.36 ± 4.81**	18.35 ± 1.04**	3.81 ± 0.12**	6.5 ± 0.17
aaBBdd	18	43.85 ± 8.16	48.05 ± 3.67**	18.17 ± 0.74**	3.8 ± 0.1**	6.47 ± 0.17
AAbbdd	48	49.24 ± 6.25*	43.11 ± 4.38*	17.08 ± 1.1**	3.62 ± 0.15***	6.39 ± 0.16

All data are shown as mean ± standard deviation. The phenotype data of each genotype were compared to that of genotype AABBDD using the two-tailed Student’s t test; *significant at 0.01 < *P* < 0.05, **significant at 0.001 < *P* < 0.01, ***significant at 0.0001 < *P* < 0.001, ****significant at *P* < 0.0001

The trend observed for TGW in the BC_1_F_2_ population, where higher TGW was observed in the lines carrying intermediate number of knockout mutations than in the lines with all alleles being mutated or wild type, became more pronounced in the BC_1_F_3_ population. The knockouts in all *TaGS3* copies led to 7.5% reduction in TGW relative to wild type (*p*-value = 1.5 × 10^−4^, Table 1). On the contrary, in two double mutants with genotypes *aabbDD* and *aaBBdd*, we observed 7.9% (*p*-value = 6.4 × 10^−3^) and 7.2% (*p*-value = 2.4 × 10^−3^) increase in TGW, respectively, compared to the wild-type line. The third double mutant with genotype *AAbbdd*, however, showed 3.8% reduction in TGW (*p*-value = 2.5 × 10^−2^). The TGW of two single mutant lines (*AABBdd* and *aaBBDD*) showed no significant difference from that of the wild-type line. The only single-locus mutant that showed 9.5% increase in TGW (*p*-value = 1.1 × 10^−3^, Table 1) was located in *TaGS3-4A* (genotype *AAbbDD*), which is also the homoeolog that is expressed at the highest level in developing grain (Zhang et al. 2020).

The relationships observed between the genotypes of mutated *TaGS3* and TGW were also similar to those observed for GW and GA (Table 1, Fig. 2b). The homozygous triple mutant *aabbdd* showed 3.4% reduction in GW (*p*-value = 4.2 × 10^−4^) and 7.2% reduction in GA (*p*-value = 1.9 × 10^−6^) compared to wild-type line. The double homozygous mutants *aabbDD* and *aaBBdd* showed 2.6% (*p*-value = 2.4 × 10^−2^) and 2.2% (*p*-value = 2.4 × 10^−2^) increase in GW, respectively. A single-genome mutant of the *TaGS3-4A* locus (*AAbbDD*) showed 2.4% increase in GW (*p*-value = 2.9 × 10^−2^). The 4.3% (*p*-value = 4.2 × 10^−2^) and 3.6% increase in GA (*p*-value = 4.2 × 10^−2^) was observed in double mutants *aabbDD* and *aaBBdd*. A significant 5.0% increase in GA (*p*-value = 1.1 × 10^−2^) was detected in single-genome mutant *AAbbDD*. Similar to the results obtained in the BC_1_F_2_ population, the *TaGS3* gene editing had no detectable effects on grain number per spike.

### The effects of TaGS3 gene editing on yield component traits in the T_5_ generation population

**Fig. 3:**
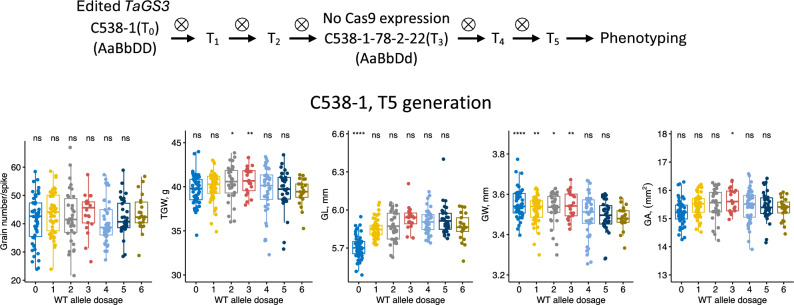
Phenotypic evaluation of *TaGS3* gene editing in the T_5_ generation population. The population was derived from the C538-1 transgenic line. The phenotypic measurements were taken using lines grouped based on the total number of wild-type (WT) alleles at three *TaGS3* loci located on chromosomes 7A, 4A and 7D. The boxplots show relationships between the GN, GW, GL, GA and TGW, and the number of functional *TaGS3* alleles. The significance testing was performed by applying post hoc *t*-test to compare each genotypic group with a reference group that includes lines with the highest number of functional wild-type alleles in each population. Significance levels: **** − ≤ 0.0001, *** − ≤ 0.001, ** − ≤ 0.01, * − ≤ 0.05, ns − > 0.05

In addition to evaluation of populations derived from the 4906-1-6 × cv. Bobwhite cross, the phenotypic effects of *TaGS3* editing were validated in the T_5_ generation population derived from T_0_ transgenic plant C538-1. In the T_0_ generation, this plant had only two of the six alleles of *TaGS3* mutated (genotype *AaBaDD*, Fig. 3). The analysis of three T_1_ lines derived from C538-1 (C538-1-1, C538-1-10 and C538-1-18) showed that this family segregates for the presence/absence of the Cas9 construct (Fig. 1c and Supplementary Fig. 1a). The qRT-PCR analysis indicated that the Cas9 transgene expression in the Cas9-positive C538-1-1 line from this family is six times higher than the expression level of actin control (Supplementary Fig. 1a). The transgenerational activity of this Cas9 transgene (Wang et al. 2018a) can therefore be used to recover new *TaGS3* mutants in the following generations. The screening of multiple lines derived from C538-1 was performed by next-generation sequencing of the pooled bar-coded amplicons generated for the *TaGS3* gRNA targets (Supplementary Table 4). In the T_3_ generation of this line, we have identified the Cas9-negative line C538-1-78-2-22, which was heterozygous at all homoeologous loci, *TaGS3-7A*, *TaGS3-4A* and *TaGS3-7D* (Fig. 1c). This line was used to develop a T_5_ generation population including 251 lines that segregated for wild-type and LOF homeo-alleles at all three *TaGS3* loci, with variation in the dosage of functional wild-type *TaGS3* alleles ranging from 0 to 6 (Fig. 3). Due to relatively small number of lines in some of the genotype classes (Supplementary Table 5), lines were grouped based on the dosage of the functional non-edited *TaGS3* alleles (6 groups) and used to investigate the relationship between the wild-type allele dosage and variation in yield component traits.

Consistent with the results obtained in the populations derived from the transgenic line 4906-1-6 (Fig. 2, Table 1), the group of lines in T_5_ population with all *TaGS3* alleles edited showed 2.8% reduction in GL compared to the wild-type lines (*p*-value = 1.2 × 10^−6^). Similar to the results obtained for the 4906-1-6-derived populations, we have observed non-additive allele dosage effects for TGW and GA with highest increase observed in lines carrying the intermediate number of functional wild-type *TaGS3* alleles (Fig. 3, Table 2). While no significant differences in TGW and GA were observed between the groups that had all *TaGS3* copies either edited or non-edited, the group of lines with three copies of the functional *TaGS3* allele showed 3.4% increase in TGW (*p*-value = 9.1 × 10^−3^) and 2.1% increase in GA (*p*-value = 1.9 × 10^−2^) compared to the wild-type lines. Thus, the decrease or increase in the total number of functional *TaGS3* alleles below or above three, respectively, was associated with the decrease in TGW and GA.

**Table 2:** Effects of CRISPR-Cas9-induced mutations in the *TaGS3* gene on grain weight, grain morphometric parameters and grain number per head in T_5_ generation population derived from the T_0_ transgenic line C581-1

Wild-type dosage	*N*	GNH	TGW (g)	GA (mm^2^)	GW (mm)	GL (mm)
0	51	41.44 ± 8.48	39.70 ± 1.70	15.22 ± 0.44	3.55 ± 0.07****	5.71 ± 0.09****
1	52	43.78 ± 7.38	40.02 ± 1.62	15.47 ± 0.38	3.53 ± 0.07**	5.85 ± 0.08
2	34	42.52 ± 9.55	40.43 ± 1.98*	15.51 ± 0.54	3.53 ± 0.08*	5.87 ± 0.12
3	20	43.90 ± 7.00	40.73 ± 1.61**	15.66 ± 0.42*	3.55 ± 0.07**	5.93 ± 0.10
4	41	40.67 ± 7.65	39.59 ± 2.77	15.45 ± 0.59	3.50 ± 0.11	5.91 ± 0.10
5	32	42.23 ± 6.69	39.64 ± 2.28	15.44 ± 0.52	3.49 ± 0.08	5.92 ± 0.12
6	21	43.76 ± 6.51	39.40 ± 1.50	15.34 ± 0.40	3.48 ± 0.06	5.87 ± 0.11

All data are shown as mean ± standard deviation. The phenotype data of each genotype were compared to that of genotype AABBDD with 6 functional wild-type homeo-alleles of *TaGS3* using the two-tailed Student’s *t*-test; *significant at 0.01 < *P* < 0.05, **significant at 0.001 < *P* < 0.01, ***significant at 0.0001 < *P* < 0.001, ****significant at *P* < 0.0001

In the T_5_ population, GW showed a trend toward increase with a decrease in the number of the functional *TaGS3* alleles (Fig. 3 and Table 2). However, this trend was not supported by the observations made in the BC_1_F_2_ and BC_1_F_3_ populations derived from the 4906-1-6 × cv. Bobwhite cross (Fig. 2), and likely could be attributed to genotype-specific epigenetic modifications associated with the regeneration of the C538-1 transgenic plant. In our previous studies, we found that it is important to take this factor into account and perform phenotypic evaluation of transgenic lines in advanced generations after crossing with wild-type line (Wang et al. 2018b).

Overall, the phenotypic effects of *TaGS3* gene editing were consistent for GL, TGW and GA in two advanced generation populations derived from independently developed transgenic lines 4906-1-6 and C538-1. Here, we showed that 1) fixation of all three *TaGS3* loci for the LOF homeo-alleles results in the reduction of GL, TGW and GA, 2) optimal expression of GL, TGW and GA was observed in the edited lines with the intermediate dosage of LOF homeo-alleles, and 3) only some of the single- and double-genome *TaGS3* knockouts in homozygous state (*AAbbDD*, *aaBBdd*, *aabbDD*) showed significant positive effect on the GL, TGW and GA traits suggestive of epistatic interaction between the edited and non-edited homeo-alleles of *TaGS3*.

## Discussion

The analyses of *TaGS3* gene sequence variation in wheat population (Yang et al. 2019b; Zhang et al. 2020; Ren et al. 2021) and functional and diversity studies of *GS3* in rice (Fan et al. 2006; Takano-Kai et al. 2009; Sun et al. 2018) suggest that the *GS3* homologs in both crops act as negative regulators of grain size and weight. However, the effects of LOF mutations in *GS3* homologs on grain size and weight in wheat and rice are different. Contrary to the results obtained in rice (Sun et al. 2018), where the LOF alleles of *GS3* are associated with highest increase in grain length and weight, the fixation of the *TaGS3* gene loci from three wheat genomes for the LOF homeo-alleles results in significant decrease in grain length, grain size and grain weight. These results suggest that the functionality of some of the *TaGS3* homoeologs is important for optimal expression of grain dimension and weight traits and indicate that the *GS3* homologs of wheat and rice are likely functionally diverged. The requirement for the presence of both functional and LOF homeo-alleles is supported by regulatory effects of *TaGS3* on grain weight and dimension. We found that the most substantial positive effects on these traits were observed in edited lines carrying three LOF homeo-alleles of the *TaGS3* gene. The effects of *TaGS3* gene editing were dosage-dependent but non-additive, suggesting that the accumulation of LOF homeo-alleles has a positive effect only up to certain levels. This indicates that *TaGS3* expression is still required for the optimal expression of grain-related traits in wheat.

The positive effects of *TaGS3* editing on grain size and weight were associated with specific wheat genomes. We found that combinations of LOF homeo-alleles at the *TaGS3-7A/TaGS3-4A* and *TaGS3-7A/TaGS3-7D* loci significantly increased grain weight and dimensions. However, the LOF allele combination at the *TaGS3-4A/TaGS3-7D* loci had a negative effect on these traits. Genetic diversity analyses in wheat showed a significant association between increased TGW and GL and the presence of two specific *TaGS3* haplotypes on chromosomes 4A and 7D, suggesting an epistatic interaction between these homoeologous gene copies (Zhang et al. 2020). It is possible that the negative phenotypic impact of LOF alleles at the *TaGS3-4A* and *TaGS3-7D* loci is associated with reduced epistasis between these homoeologous gene loci.

Our results indicate that each of the three homoeologous copies of *TaGS3* is functional and has potential to influence grain size and weight traits in wheat. This observation is consistent with the analyses of natural genetic diversity that linked variation in all three *TaGS3* gene copies from chromosomes 7A, 4A and 7D with variation in grain size and weight in wheat (Yang et al. 2019b; Zhang et al. 2020). The differences in the phenotypic impact of editing distinct homoeologous copies of *TaGS3* could potentially be associated with cultivar- and genome-specific differences in expression and alternative splicing. Previously, we have observed cultivar-specific phenotypic effects of editing distinct homoeologous copies of *TaGW2* on grain size and weight that are associated with gene expression levels in different cultivars (Wang et al. 2018b). The effects of alternative splicing in *GS3* isoforms and grain size and weight traits was demonstrated for rice (Liu et al. 2022), with two detected *GS3* isoforms having the suppressive OSR domain and negatively impacting grain size and weight (Liu et al. 2022). In wheat, five *TaGS3* isoforms were detected for each of the three *TaGS3* homoeologs (Ren et al. 2021). Overexpression of the rarest *TaGS3* isoforms (*TaGS3.5*) representing only 0.3% of all transcripts in cv. ‘Kenong 199’ and cv. ‘Kenong 9204,’ and carrying the truncated OSR domain had strong positive impact on grain size and weight (Ren et al. 2021). However, since this isoform represents only minor fraction of all *TaGS3* transcripts, it is unlikely that changes in *TaGS3.5* abundance could explain phenotypic effects of *TaGS3* mutations observed in our study. First, the levels of *TaGS3.5* expression could not be reliably detected using RT-PCR in the spikes of cv. ‘Bobwhite.’ Second, the analyses of other *TaGS3* isoforms in our mutants showed no statistically significant changes compared to wild-type cv. ‘Bobwhite’ (Supplementary Table 6). These results combined with observation that the *TaGS3* homoeolog expression levels in cv. ‘Bobwhite’ are nearly identical (Supplementary Fig. 1b) suggest that the effects of *TaGS3* editing on grain size and weight in our study are unlikely associated the changes in the isoform or homeo-allele abundance, warranting further research into the genetic control of grain-related traits by *TaGS3* in wheat.

In summary, our work provides further insights into the complex genetic control of grain dimension and weight traits by the *TaGS3* homoeologous loci in wheat. Our results indicate that the wheat homologs of the rice *GS3* gene act not only as negative regulators of grain length and weight. Contrary to the *GS3* effects in rice, the presence of a certain number of functional *TaGS3* gene copies appears to be critical for optimal expression of grain morphometric and weight traits in wheat. Using CRISPR-Cas9 editing, we developed LOF homeo-alleles for *TaGS3* and identified single- and two-locus combinations of these alleles that have the potential to increase grain length and weight. Combined with the *TaGS3* alleles identified in natural populations, these LOF alleles broaden genetic diversity available for improving yield potential in wheat breeding programs.

## Supplementary Information

Below is the link to the electronic supplementary material.

Supplementary file1 (DOCX 246 KB)

## Data Availability

CRISPR-Cas9 gene editing constructs and wheat lines are available upon request.
