# Correction: Non‑additive dosage‑dependent effects of TaGS3 gene editing on grain size and weight in wheat

**DOI:** 10.1007/s00122-025-04998-6

**Published:** 2025-08-19

**Authors:** Wei Wang, Qianli Pan, Bin Tian, Zitong Yu, Dwight Davidson, Guihua Bai, Alina Akhunova, Harold Trick, Eduard Akhunov

**Affiliations:** 1 Wheat Genetics Resource Center, Kansas State University, Manhattan, KS USA; 2 Nanjing Agricultural University, Nanjing, China; 3 USDA-ARS Hard Winter Wheat Genetics Research Unit, Manhattan, KS USA; 4 Integrated Genomics Facility, Kansas State University, Manhattan, KS USA

**Correction to: Theoretical and Applied Genetics (2025) 138:38** 10.1007/s00122-025-04827-w

The figures 2b and 3 had missing labels on the X- and Y-axes in the published article.

The corrected Figs. 2b and 3 are provided below.

**Fig. 2:**
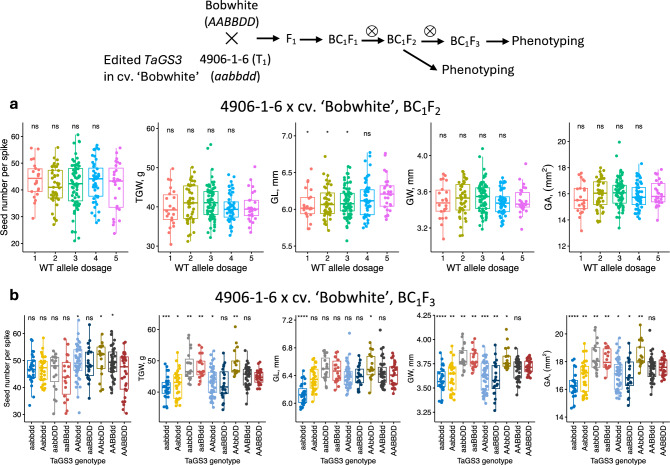
Efects of TaGS3 gene editing on yield component traits assessed in the BC1F2 and BC1F3 populations. **a** The BC1F2 population was created by crossing 4906-1-6 line with wild-type cultivar Bobwhite. The phenotypic measurements were taken using lines grouped based on the total number of functional wild-type (WT) TaGS3 alleles at three loci located on chromosomes 7A, 4A and 7D. **b** The phenotypic measurements were taken using the lines grouped based on their genotypes at three TaGS3 homoeologous loci. The upper- and lowercase letters correspond to genotypes of the functional (A, B or D) and edited (a, b or d) TaGS3 alleles, respectively. The graphs show relationships between grain number, morphometric traits and weight and the number of functional wild-type TaGS3 alleles. The signifcance testing was performed by applying t-test to compare each genotype group with a reference group that includes lines with the highest number of functional wild-type alleles in each population. Signifcance levels: **** −  ≤ 0.0001, *** −  ≤ 0.001, ** −  ≤ 0.01, * −  ≤ 0.05, ns −  > 0.05

**Fig. 3:**
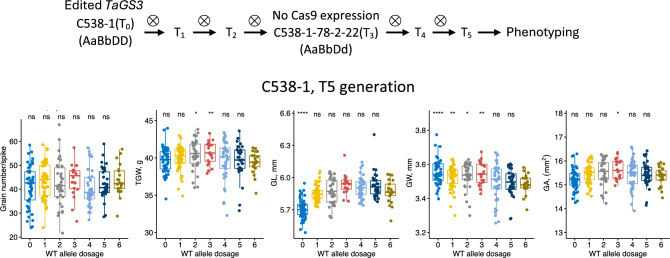
Phenotypic evaluation of TaGS3 gene editing in the T5 generation population. The population was derived from the C538-1 transgenic line. The phenotypic measurements were taken using lines grouped based on the total number of wild-type (WT) alleles at three TaGS3 loci located on chromosomes 7A, 4A and 7D. The boxplots show relationships between the GN, GW, GL, GA and TGW, and the number of functional TaGS3 alleles. The signifcance testing was performed by applying post hoc t-test to compare each genotypic group with a reference group that includes lines with the highest number of functional wild-type alleles in each population. Signifcance levels: **** −  ≤ 0.0001, *** −  ≤ 0.001, ** −  ≤ 0.01, * −  ≤ 0.05, ns −  > 0.05

The original article has been corrected.

