# Supplementary material for: Non-additive dosage-dependent effects of TaGS3 gene editing on grain size and weight in wheat
Source: Theor Appl Genet. 2025 Jan 29;138(2):38. doi: 10.1007/s00122-025-04827-w (PMC11779757; doi:10.1007/s00122-025-04827-w)
Supplement: Supplementary file 1 — Supplementary file1 (DOCX 246 KB) [file 122_2025_4827_MOESM1_ESM.docx]

**Supplementary Materials**

**Supplementary Figures**

**Supplementary Fig. 1. A.** *Cas9* expression levels in a set of T_1_ generation lines derived from the transgenic lines C538-1 and 4906-1. The expression level is shown relative to the *Actin* gene expression level. BW corresponds to wild-type cultivar ‘Bobwhite’. No detectable levels of *Cas9* expression are found in line C538-1-1, C538-1-10, C538-1-18, 4906-1-8, 4906-1-2 and 4906-1-1. **B.** The mean expression levels (± standard errors) of the three *TaGS3* gene homoeologs based on RNA-seq reads from the spikes of wild-type cultivar ‘Bobwhite’. The mean and standard errors are based on three replicates. The expression values correspond to log2-transformed read counts that were subjected to variance stabilizing transformation using the “*vst*” function of DESeq2, removing the dependence of the expression variance on the mean.

**Supplementary Tables**

**Supplementary Table 1.** List of oligos used in the project.

| **Oligo name** | **Sequence** | **Usage** |
| --- | --- | --- |
| GS3T6-9checkF | TCCATTGATGACGCTCTCTG | PCR primer |
| GS3T6-9checkR | TCGAGGAAGCTGATCTGG | PCR primer |
| TaGS3T6F | cttGGCCGGCAATGGCGGCGCCC | gRNA oligo |
| TaGS3T6R | aaacGGCCGGCAATGGCGGCGCC | gRNA oligo |
| TaGS3T7F | cttgAAGTCCCCGCTCGACCCCTG | gRNA oligo |
| TaGS3T7R | aaacCAGGGGTCGAGCGGGGACTT | gRNA oligo |
| TaGS3T8F | cttGCGGCCGCAGGGGTCGAGCG | gRNA oligo |
| TaGS3T8R | aaacCGCTCGACCCCTGCGGCCG | gRNA oligo |
| TaGS3T9F | cttGCAGCCGGTGGCGGCCGCAG | gRNA oligo |
| TaGS3T9R | aaacCTGCGGCCGCCACCGGCTG | gRNA oligo |

**Supplementary Table 2.** Phenotypic traits collected for BC_1_F_2_ population derived from 4906-1-6. Traits collected include grain number per head (GNH), thousand grain weight (TGW), grain area (GA), grain width (GW) and grain length (GL). “A”, “B” and “D” designations are given to the wild-type alleles, and “a”, “b” and “d designations are given to the mutant alleles.

| **ID** | **Dosage** | **Genotype** | **GNH** | **TGW (g)** | **GA (mm^2^)** | **GW (mm)** | **GL (mm)** |
| --- | --- | --- | --- | --- | --- | --- | --- |
| YLD12BC1F1(1)-1-13 | 1 | aaBbdd | 47.00 | 38.23 | 15.12 | 3.38 | 5.95 |
| YLD12BC1F1(1)-1-26 | 1 | aabbDd | 38.33 | 49.74 | 17.07 | 3.78 | 6.00 |
| YLD12BC1F1(1)-1-33 | 1 | aabbDd | 37.33 | 43.30 | 16.42 | 3.63 | 6.01 |
| YLD12BC1F1(1)-1-36 | 1 | Aabbdd | 55.33 | 39.58 | 15.49 | 3.52 | 5.80 |
| YLD12BC1F1(1)-1-4 | 1 | Aabbdd | 40.33 | 43.88 | 16.99 | 3.73 | 6.27 |
| YLD12BC1F1(1)-1-5 | 1 | aabbDd | 51.33 | 42.14 | 16.38 | 3.59 | 6.06 |
| YLD12BC1F1(1)-1-73 | 1 | aabbDd | 42.33 | 44.88 | 17.26 | 3.74 | 6.38 |
| YLD12BC1F1(1)-1-87 | 1 | Aabbdd | 39.67 | 35.88 | 15.04 | 3.41 | 6.02 |
| YLD12BC1F1(1)-1-89 | 1 | aabbDd | 36.00 | 46.85 | 17.58 | 3.80 | 6.39 |
| YLD12BC1F1(1)-5-127 | 1 | aabbDd | 35.00 | 40.19 | 15.76 | 3.51 | 6.04 |
| YLD12BC1F1(1)-5-133 | 1 | aaBbdd | 46.00 | 39.49 | 16.40 | 3.50 | 6.55 |
| YLD12BC1F1(1)-5-141 | 1 | Aabbdd | 49.00 | 34.63 | 14.51 | 3.31 | 5.90 |
| YLD12BC1F1(1)-5-151 | 1 | aabbDd | 42.67 | 37.19 | 15.22 | 3.39 | 6.15 |
| YLD12BC1F1(1)-5-18 | 1 | aabbDd | 48.00 | 30.42 | 13.17 | 3.08 | 5.73 |
| YLD12BC1F1(1)-5-29 | 1 | aaBbdd | 39.33 | 37.63 | 14.72 | 3.31 | 6.03 |
| YLD12BC1F1(1)-5-50 | 1 | aaBbdd | 45.67 | 35.04 | 14.84 | 3.31 | 6.17 |
| YLD12BC1F1(1)-5-56 | 1 | aaBbdd | 29.33 | 47.05 | 17.47 | 3.74 | 6.29 |
| YLD12BC1F1(1)-5-5 | 1 | aabbDd | 55.67 | 38.92 | 15.51 | 3.45 | 5.99 |
| YLD12BC1F1(1)-5-79 | 1 | Aabbdd | 51.67 | 33.42 | 14.19 | 3.24 | 5.94 |
| YLD12BC1F1(1)-5-86 | 1 | aaBbdd | 47.67 | 42.38 | 16.27 | 3.59 | 6.01 |
| YLD12BC1F1(1)-5-89 | 1 | aabbDd | 45.33 | 38.09 | 15.29 | 3.45 | 5.93 |
| YLD12BC1F1(1)-5-8 | 1 | aabbDd | 43.33 | 36.62 | 14.59 | 3.40 | 5.74 |
| YLD12BC1F1(1)-1-14 | 2 | aaBbDd | 28.67 | 43.49 | 16.09 | 3.70 | 5.71 |
| YLD12BC1F1(1)-1-15 | 2 | aaBbDd | 40.67 | 46.56 | 17.18 | 3.75 | 6.07 |
| YLD12BC1F1(1)-1-18 | 2 | AabbDd | 38.67 | 47.67 | 17.28 | 3.79 | 6.07 |
| YLD12BC1F1(1)-1-27 | 2 | aaBbDd | 34.33 | 50.10 | 17.39 | 3.76 | 6.22 |
| YLD12BC1F1(1)-1-2 | 2 | aabbDD | 39.00 | 47.44 | 17.99 | 3.82 | 6.46 |
| YLD12BC1F1(1)-1-32 | 2 | aaBBdd | 37.00 | 45.50 | 17.49 | 3.72 | 6.35 |
| YLD12BC1F1(1)-1-35 | 2 | AabbDd | 39.00 | 44.36 | 16.69 | 3.54 | 6.29 |
| YLD12BC1F1(1)-1-40 | 2 | AaBbdd | 48.67 | 35.75 | 14.50 | 3.30 | 5.80 |
| YLD12BC1F1(1)-1-41 | 2 | AabbDd | 42.33 | 48.90 | 17.78 | 3.75 | 6.33 |
| YLD12BC1F1(1)-1-43 | 2 | AaBbdd | 36.33 | 45.60 | 16.81 | 3.74 | 5.97 |
| YLD12BC1F1(1)-1-45 | 2 | AabbDd | 38.00 | 46.67 | 17.15 | 3.71 | 6.13 |
| YLD12BC1F1(1)-1-50 | 2 | aaBbDd | 45.33 | 42.06 | 16.22 | 3.57 | 6.00 |
| YLD12BC1F1(1)-1-69 | 2 | AabbDd | 38.00 | 44.56 | 17.28 | 3.70 | 6.48 |
| YLD12BC1F1(1)-1-72 | 2 | AaBbdd | 38.33 | 46.96 | 17.69 | 3.82 | 6.34 |
| YLD12BC1F1(1)-1-83 | 2 | aaBbDd | 51.33 | 38.70 | 15.93 | 3.44 | 6.38 |
| YLD12BC1F1(1)-1-92 | 2 | aaBbDd | 39.33 | 45.51 | 16.30 | 3.63 | 5.95 |
| YLD12BC1F1(1)-1-94 | 2 | aaBbDd | 27.00 | 41.36 | 15.34 | 3.52 | 5.79 |
| YLD12BC1F1(1)-1-95 | 2 | aaBbDd | 37.00 | 45.68 | 16.64 | 3.66 | 5.99 |
| YLD12BC1F1(1)-5-103 | 2 | AaBbdd | 31.00 | 37.85 | 15.32 | 3.42 | 6.07 |
| YLD12BC1F1(1)-5-126 | 2 | AabbDd | 45.33 | 37.06 | 15.40 | 3.42 | 6.07 |
| YLD12BC1F1(1)-5-129 | 2 | aaBbDd | 43.00 | 45.35 | 16.92 | 3.68 | 6.14 |
| YLD12BC1F1(1)-5-131 | 2 | AabbDd | 52.00 | 40.00 | 15.67 | 3.46 | 6.03 |
| YLD12BC1F1(1)-5-140 | 2 | AaBbdd | 47.33 | 31.20 | 13.92 | 3.11 | 5.98 |
| YLD12BC1F1(1)-5-14 | 2 | AAbbdd | 44.33 | 36.69 | 15.42 | 3.42 | 6.30 |
| YLD12BC1F1(1)-5-159 | 2 | aaBbDd | 36.00 | 32.59 | 13.83 | 3.21 | 5.83 |
| YLD12BC1F1(1)-5-27 | 2 | aabbDD | 48.67 | 40.89 | 16.25 | 3.59 | 6.05 |
| YLD12BC1F1(1)-5-28 | 2 | aaBbDd | 37.67 | 40.71 | 16.07 | 3.48 | 6.16 |
| YLD12BC1F1(1)-5-33 | 2 | aaBbDd | 41.67 | 39.44 | 15.43 | 3.49 | 5.86 |
| YLD12BC1F1(1)-5-34 | 2 | AabbDd | 36.67 | 36.73 | 14.75 | 3.38 | 5.86 |
| YLD12BC1F1(1)-5-35 | 2 | AAbbdd | 45.00 | 42.22 | 16.17 | 3.58 | 6.20 |
| YLD12BC1F1(1)-5-43 | 2 | aaBbDd | 49.67 | 41.01 | 16.05 | 3.55 | 5.97 |
| YLD12BC1F1(1)-5-47 | 2 | AaBbdd | 46.33 | 37.70 | 15.18 | 3.38 | 5.92 |
| YLD12BC1F1(1)-5-48 | 2 | AaBbdd | 39.00 | 37.35 | 15.07 | 3.40 | 5.94 |
| YLD12BC1F1(1)-5-49 | 2 | AabbDd | 49.00 | 41.36 | 16.95 | 3.56 | 6.72 |
| YLD12BC1F1(1)-5-51 | 2 | AaBbdd | 44.33 | 36.39 | 15.18 | 3.39 | 6.15 |
| YLD12BC1F1(1)-5-55 | 2 | aaBBdd | 42.67 | 42.19 | 16.22 | 3.60 | 6.01 |
| YLD12BC1F1(1)-5-68 | 2 | AabbDd | 47.33 | 33.45 | 14.60 | 3.27 | 6.19 |
| YLD12BC1F1(1)-5-70 | 2 | AabbDd | 50.00 | 43.73 | 17.08 | 3.66 | 6.40 |
| YLD12BC1F1(1)-5-73 | 2 | AaBbdd | 48.67 | 38.15 | 15.69 | 3.47 | 6.22 |
| YLD12BC1F1(1)-5-77 | 2 | AabbDd | 48.67 | 35.96 | 14.67 | 3.32 | 5.86 |
| YLD12BC1F1(1)-5-85 | 2 | AabbDd | 29.33 | 41.70 | 16.00 | 3.53 | 6.10 |
| YLD12BC1F1(1)-5-87 | 2 | aaBBdd | 41.00 | 39.92 | 15.58 | 3.47 | 5.94 |
| YLD12BC1F1(1)-5-88 | 2 | aaBbDd | 56.00 | 36.73 | 14.94 | 3.36 | 5.92 |
| YLD12BC1F1(1)-5-91 | 2 | aaBbDd | 29.00 | 32.87 | 13.89 | 3.12 | 5.96 |
| YLD12BC1F1(1)-5-96 | 2 | AAbbdd | 36.67 | 36.09 | 14.50 | 3.30 | 5.93 |
| YLD12BC1F1(1)-1-10 | 3 | AaBbDd | 48.00 | 45.63 | 16.98 | 3.77 | 5.91 |
| YLD12BC1F1(1)-1-16 | 3 | AABbdd | 60.67 | 40.00 | 15.81 | 3.47 | 6.02 |
| YLD12BC1F1(1)-1-17 | 3 | AaBbDd | 35.33 | 32.17 | 13.38 | 3.19 | 5.57 |
| YLD12BC1F1(1)-1-21 | 3 | AaBbDd | 49.33 | 44.59 | 16.93 | 3.65 | 6.17 |
| YLD12BC1F1(1)-1-22 | 3 | AaBbDd | 38.67 | 47.67 | 17.19 | 3.76 | 6.03 |
| YLD12BC1F1(1)-1-25 | 3 | aaBBDd | 52.00 | 39.04 | 15.13 | 3.45 | 5.82 |
| YLD12BC1F1(1)-1-30 | 3 | AaBbDd | 43.67 | 45.50 | 16.34 | 3.64 | 5.98 |
| YLD12BC1F1(1)-1-31 | 3 | AABbdd | 24.33 | 55.89 | 19.95 | 4.08 | 6.49 |
| YLD12BC1F1(1)-1-34 | 3 | AABbdd | 40.67 | 45.25 | 16.56 | 3.64 | 6.02 |
| YLD12BC1F1(1)-1-37 | 3 | AaBbDd | 21.00 | 43.65 | 16.09 | 3.63 | 5.89 |
| YLD12BC1F1(1)-1-42 | 3 | AaBbDd | 38.33 | 43.48 | 16.41 | 3.62 | 6.01 |
| YLD12BC1F1(1)-1-44 | 3 | AaBbDd | 49.33 | 45.20 | 17.28 | 3.68 | 6.18 |
| YLD12BC1F1(1)-1-52 | 3 | AAbbDd | 24.33 | 54.93 | 18.90 | 3.99 | 6.29 |
| YLD12BC1F1(1)-1-53 | 3 | AaBbDd | 47.33 | 45.63 | 17.36 | 3.69 | 6.21 |
| YLD12BC1F1(1)-1-55 | 3 | AABbdd | 40.00 | 49.58 | 17.80 | 3.85 | 6.13 |
| YLD12BC1F1(1)-1-61 | 3 | aaBbDD | 49.00 | 40.48 | 16.29 | 3.52 | 6.28 |
| YLD12BC1F1(1)-1-63 | 3 | aaBBDd | 35.00 | 43.24 | 16.00 | 3.61 | 5.87 |
| YLD12BC1F1(1)-1-65 | 3 | aaBbDD | 38.00 | 43.95 | 16.60 | 3.66 | 6.03 |
| YLD12BC1F1(1)-1-66 | 3 | AaBbDd | 37.00 | 37.57 | 14.55 | 3.38 | 5.77 |
| YLD12BC1F1(1)-1-6 | 3 | AAbbDd | 34.67 | 44.90 | 16.61 | 3.66 | 6.03 |
| YLD12BC1F1(1)-1-70 | 3 | aaBBDd | 50.33 | 41.06 | 16.49 | 3.57 | 6.37 |
| YLD12BC1F1(1)-1-71 | 3 | AabbDD | 37.33 | 46.34 | 17.62 | 3.80 | 6.37 |
| YLD12BC1F1(1)-1-77 | 3 | AAbbDd | 46.00 | 41.45 | 17.06 | 3.67 | 6.35 |
| YLD12BC1F1(1)-1-79 | 3 | AaBbDd | 36.00 | 44.35 | 16.90 | 3.66 | 6.43 |
| YLD12BC1F1(1)-1-80 | 3 | AaBbDd | 38.33 | 36.96 | 15.25 | 3.37 | 6.24 |
| YLD12BC1F1(1)-1-81 | 3 | AAbbDd | 31.33 | 44.04 | 16.77 | 3.70 | 6.21 |
| YLD12BC1F1(1)-1-82 | 3 | aaBBDd | 33.33 | 46.30 | 17.67 | 3.77 | 6.43 |
| YLD12BC1F1(1)-1-91 | 3 | aaBBDd | 34.33 | 41.75 | 16.33 | 3.59 | 6.08 |
| YLD12BC1F1(1)-1-93 | 3 | AaBbDd | 28.33 | 43.76 | 16.18 | 3.62 | 5.88 |
| YLD12BC1F1(1)-1-96 | 3 | AaBbDd | 32.67 | 43.67 | 16.74 | 3.67 | 6.06 |
| YLD12BC1F1(1)-5-100 | 3 | AaBbDd | 41.00 | 38.21 | 15.19 | 3.39 | 5.94 |
| YLD12BC1F1(1)-5-101 | 3 | AaBbDd | 34.33 | 34.56 | 14.18 | 3.31 | 5.75 |
| YLD12BC1F1(1)-5-106 | 3 | aaBBDd | 48.33 | 36.97 | 14.99 | 3.27 | 6.12 |
| YLD12BC1F1(1)-5-107 | 3 | AaBBdd | 42.00 | 43.89 | 17.35 | 3.71 | 6.49 |
| YLD12BC1F1(1)-5-109 | 3 | AAbbDd | 45.33 | 36.91 | 15.01 | 3.33 | 5.99 |
| YLD12BC1F1(1)-5-10 | 3 | AABbdd | 47.00 | 44.04 | 16.65 | 3.59 | 6.14 |
| YLD12BC1F1(1)-5-116 | 3 | aaBbDD | 45.33 | 34.85 | 14.73 | 3.31 | 5.99 |
| YLD12BC1F1(1)-5-117 | 3 | aaBbDD | 44.67 | 37.76 | 15.06 | 3.41 | 5.85 |
| YLD12BC1F1(1)-5-124 | 3 | AaBbDd | 34.00 | 40.59 | 16.05 | 3.46 | 6.22 |
| YLD12BC1F1(1)-5-125 | 3 | AaBbDd | 45.00 | 37.48 | 15.63 | 3.41 | 6.08 |
| YLD12BC1F1(1)-5-132 | 3 | AaBbDd | 48.00 | 38.06 | 16.04 | 3.47 | 6.58 |
| YLD12BC1F1(1)-5-134 | 3 | AAbbDd | 46.67 | 38.86 | 15.50 | 3.37 | 6.19 |
| YLD12BC1F1(1)-5-136 | 3 | AaBBdd | 51.33 | 40.00 | 16.27 | 3.51 | 6.40 |
| YLD12BC1F1(1)-5-139 | 3 | AaBbDd | 45.00 | 36.81 | 15.08 | 3.37 | 5.98 |
| YLD12BC1F1(1)-5-13 | 3 | AaBBdd | 57.67 | 39.77 | 15.77 | 3.48 | 6.03 |
| YLD12BC1F1(1)-5-143 | 3 | AaBbDd | 49.00 | 39.05 | 15.88 | 3.42 | 6.24 |
| YLD12BC1F1(1)-5-149 | 3 | AaBbDd | 58.33 | 35.43 | 14.75 | 3.32 | 5.89 |
| YLD12BC1F1(1)-5-150 | 3 | AaBBdd | 49.67 | 38.05 | 15.26 | 3.45 | 5.93 |
| YLD12BC1F1(1)-5-160 | 3 | AAbbDd | 42.67 | 33.44 | 14.20 | 3.23 | 5.89 |
| YLD12BC1F1(1)-5-16 | 3 | aaBbDD | 46.33 | 41.80 | 16.54 | 3.59 | 6.11 |
| YLD12BC1F1(1)-5-17 | 3 | aaBbDD | 49.00 | 36.12 | 14.92 | 3.35 | 5.97 |
| YLD12BC1F1(1)-5-20 | 3 | AaBbDd | 48.00 | 38.75 | 15.68 | 3.45 | 6.04 |
| YLD12BC1F1(1)-5-21 | 3 | AaBbDd | 41.67 | 36.56 | 14.90 | 3.37 | 5.87 |
| YLD12BC1F1(1)-5-24 | 3 | AABbdd | 41.67 | 39.76 | 15.66 | 3.53 | 5.90 |
| YLD12BC1F1(1)-5-26 | 3 | aaBBDd | 39.33 | 41.78 | 16.19 | 3.55 | 6.12 |
| YLD12BC1F1(1)-5-2 | 3 | AAbbDd | 50.67 | 41.05 | 15.76 | 3.45 | 6.14 |
| YLD12BC1F1(1)-5-31 | 3 | AaBbDd | 39.00 | 42.82 | 15.85 | 3.55 | 5.98 |
| YLD12BC1F1(1)-5-36 | 3 | AaBbDd | 32.00 | 41.35 | 16.27 | 3.56 | 6.16 |
| YLD12BC1F1(1)-5-37 | 3 | AaBBdd | 45.00 | 36.74 | 14.98 | 3.37 | 5.93 |
| YLD12BC1F1(1)-5-39 | 3 | aaBbDD | 42.33 | 39.84 | 15.91 | 3.49 | 6.12 |
| YLD12BC1F1(1)-5-46 | 3 | AaBbDd | 24.33 | 38.63 | 15.92 | 3.55 | 6.07 |
| YLD12BC1F1(1)-5-53 | 3 | AaBbDd | 43.00 | 41.71 | 16.56 | 3.61 | 6.33 |
| YLD12BC1F1(1)-5-54 | 3 | aaBbDD | 35.67 | 42.90 | 16.65 | 3.59 | 6.22 |
| YLD12BC1F1(1)-5-58 | 3 | AabbDD | 42.33 | 42.83 | 16.51 | 3.55 | 6.19 |
| YLD12BC1F1(1)-5-65 | 3 | AaBbDd | 22.00 | 33.94 | 14.53 | 3.29 | 5.98 |
| YLD12BC1F1(1)-5-69 | 3 | AAbbDd | 41.00 | 41.79 | 16.03 | 3.53 | 6.04 |
| YLD12BC1F1(1)-5-71 | 3 | AabbDD | 34.00 | 39.90 | 16.06 | 3.53 | 6.28 |
| YLD12BC1F1(1)-5-75 | 3 | AabbDD | 53.67 | 42.05 | 16.74 | 3.61 | 6.36 |
| YLD12BC1F1(1)-5-82 | 3 | aaBbDD | 44.33 | 37.37 | 15.29 | 3.38 | 6.07 |
| YLD12BC1F1(1)-5-83 | 3 | AaBBdd | 44.67 | 44.70 | 17.47 | 3.68 | 6.57 |
| YLD12BC1F1(1)-5-95 | 3 | aaBbDD | 42.00 | 39.68 | 15.49 | 3.48 | 5.94 |
| YLD12BC1F1(1)-5-97 | 3 | AaBBdd | 58.00 | 40.46 | 16.20 | 3.55 | 6.21 |
| YLD12BC1F1(1)-1-11 | 4 | AABbDd | 46.33 | 40.50 | 15.84 | 3.45 | 6.13 |
| YLD12BC1F1(1)-1-1 | 4 | AABbDd | 55.00 | 38.85 | 15.89 | 3.42 | 6.24 |
| YLD12BC1F1(1)-1-20 | 4 | AaBBDd | 41.00 | 40.08 | 15.80 | 3.48 | 6.13 |
| YLD12BC1F1(1)-1-24 | 4 | AaBBDd | 31.33 | 38.40 | 14.49 | 3.37 | 5.75 |
| YLD12BC1F1(1)-1-28 | 4 | AABbDd | 35.33 | 48.21 | 16.80 | 3.66 | 6.12 |
| YLD12BC1F1(1)-1-29 | 4 | AABbDd | 55.33 | 41.08 | 15.73 | 3.44 | 6.11 |
| YLD12BC1F1(1)-1-46 | 4 | AaBbDD | 48.67 | 41.85 | 16.48 | 3.54 | 6.20 |
| YLD12BC1F1(1)-1-48 | 4 | AaBBDd | 43.67 | 39.39 | 15.59 | 3.50 | 5.90 |
| YLD12BC1F1(1)-1-49 | 4 | AABbDd | 53.00 | 39.62 | 15.71 | 3.51 | 5.89 |
| YLD12BC1F1(1)-1-68 | 4 | AABBdd | 36.67 | 45.18 | 17.56 | 3.71 | 6.66 |
| YLD12BC1F1(1)-1-74 | 4 | AaBbDD | 37.33 | 44.73 | 16.91 | 3.72 | 6.29 |
| YLD12BC1F1(1)-1-75 | 4 | AaBBDd | 28.67 | 47.67 | 18.30 | 3.71 | 6.77 |
| YLD12BC1F1(1)-1-78 | 4 | AaBbDD | 37.67 | 46.46 | 17.46 | 3.76 | 6.45 |
| YLD12BC1F1(1)-1-88 | 4 | AaBbDD | 40.00 | 38.58 | 16.07 | 3.51 | 6.40 |
| YLD12BC1F1(1)-1-9 | 4 | AAbbDD | 42.00 | 45.32 | 17.35 | 3.70 | 6.42 |
| YLD12BC1F1(1)-5-104 | 4 | AaBbDD | 50.33 | 36.62 | 14.82 | 3.38 | 5.88 |
| YLD12BC1F1(1)-5-110 | 4 | AABbDd | 30.33 | 37.69 | 15.48 | 3.36 | 6.19 |
| YLD12BC1F1(1)-5-112 | 4 | AABBdd | 45.00 | 40.37 | 16.17 | 3.53 | 6.26 |
| YLD12BC1F1(1)-5-113 | 4 | AABBdd | 43.67 | 41.07 | 16.25 | 3.55 | 6.24 |
| YLD12BC1F1(1)-5-115 | 4 | AaBBDd | 42.33 | 35.12 | 14.75 | 3.27 | 6.09 |
| YLD12BC1F1(1)-5-118 | 4 | AABbDd | 45.33 | 39.34 | 15.56 | 3.54 | 5.93 |
| YLD12BC1F1(1)-5-119 | 4 | AaBBDd | 44.33 | 36.92 | 15.52 | 3.38 | 6.29 |
| YLD12BC1F1(1)-5-12 | 4 | AaBBDd | 44.00 | 40.76 | 15.86 | 3.50 | 6.05 |
| YLD12BC1F1(1)-5-135 | 4 | AABbDd | 56.67 | 36.94 | 15.35 | 3.38 | 6.10 |
| YLD12BC1F1(1)-5-138 | 4 | AABBdd | 55.33 | 39.10 | 16.03 | 3.46 | 6.27 |
| YLD12BC1F1(1)-5-142 | 4 | AaBBDd | 52.67 | 32.72 | 14.24 | 3.22 | 5.90 |
| YLD12BC1F1(1)-5-144 | 4 | aaBBDD | 51.33 | 33.77 | 14.51 | 3.24 | 5.99 |
| YLD12BC1F1(1)-5-145 | 4 | AABbDd | 40.67 | 38.11 | 15.29 | 3.38 | 6.05 |
| YLD12BC1F1(1)-5-147 | 4 | AABBdd | 47.00 | 39.79 | 16.01 | 3.47 | 6.19 |
| YLD12BC1F1(1)-5-148 | 4 | AABbDd | 45.00 | 34.59 | 14.52 | 3.26 | 5.92 |
| YLD12BC1F1(1)-5-158 | 4 | AABbDd | 41.00 | 36.42 | 15.20 | 3.31 | 6.12 |
| YLD12BC1F1(1)-5-15 | 4 | AaBBDd | 55.67 | 39.10 | 15.74 | 3.46 | 6.03 |
| YLD12BC1F1(1)-5-1 | 4 | AaBBDd | 41.00 | 36.83 | 14.48 | 3.33 | 5.80 |
| YLD12BC1F1(1)-5-22 | 4 | AaBBDd | 45.33 | 36.76 | 14.98 | 3.33 | 6.04 |
| YLD12BC1F1(1)-5-25 | 4 | AaBBDd | 37.67 | 40.18 | 15.48 | 3.49 | 5.92 |
| YLD12BC1F1(1)-5-41 | 4 | AABbDd | 48.00 | 38.47 | 15.57 | 3.39 | 6.09 |
| YLD12BC1F1(1)-5-42 | 4 | AaBbDD | 34.67 | 45.58 | 17.33 | 3.70 | 6.24 |
| YLD12BC1F1(1)-5-45 | 4 | AaBbDD | 33.33 | 37.70 | 14.85 | 3.43 | 5.77 |
| YLD12BC1F1(1)-5-4 | 4 | AaBbDD | 41.67 | 39.12 | 15.63 | 3.52 | 5.93 |
| YLD12BC1F1(1)-5-52 | 4 | AaBBDd | 31.67 | 39.68 | 16.16 | 3.50 | 6.61 |
| YLD12BC1F1(1)-5-59 | 4 | AaBbDD | 27.67 | 34.82 | 14.09 | 3.28 | 5.81 |
| YLD12BC1F1(1)-5-67 | 4 | AABbDd | 46.00 | 40.87 | 16.78 | 3.52 | 6.72 |
| YLD12BC1F1(1)-5-6 | 4 | AaBBDd | 44.33 | 44.21 | 16.71 | 3.64 | 6.12 |
| YLD12BC1F1(1)-5-72 | 4 | AABbDd | 44.67 | 43.73 | 17.23 | 3.64 | 6.46 |
| YLD12BC1F1(1)-5-84 | 4 | aaBBDD | 49.67 | 36.24 | 15.10 | 3.43 | 6.11 |
| YLD12BC1F1(1)-5-92 | 4 | AABbDd | 44.33 | 37.52 | 14.72 | 3.36 | 5.85 |
| YLD12BC1F1(1)-5-93 | 4 | AaBbDD | 35.33 | 43.02 | 16.47 | 3.63 | 6.01 |
| YLD12BC1F1(1)-5-99 | 4 | AAbbDD | 55.00 | 43.45 | 17.05 | 3.62 | 6.36 |
| YLD12BC1F1(1)-1-19 | 5 | AABbDD | 54.00 | 38.15 | 15.42 | 3.45 | 5.92 |
| YLD12BC1F1(1)-1-51 | 5 | AABbDD | 28.33 | 50.24 | 17.86 | 3.91 | 6.07 |
| YLD12BC1F1(1)-1-84 | 5 | AABBDd | 32.67 | 43.98 | 16.87 | 3.64 | 6.31 |
| YLD12BC1F1(1)-5-105 | 5 | AABbDD | 46.67 | 38.79 | 15.76 | 3.44 | 6.16 |
| YLD12BC1F1(1)-5-108 | 5 | AABBDd | 43.33 | 36.31 | 15.09 | 3.43 | 5.90 |
| YLD12BC1F1(1)-5-111 | 5 | AaBBDD | 55.67 | 37.01 | 15.33 | 3.39 | 6.24 |
| YLD12BC1F1(1)-5-114 | 5 | AABBDd | 34.00 | 43.63 | 16.70 | 3.65 | 6.15 |
| YLD12BC1F1(1)-5-11 | 5 | AABBDd | 43.67 | 41.15 | 15.95 | 3.46 | 6.20 |
| YLD12BC1F1(1)-5-122 | 5 | AaBBDD | 50.67 | 39.74 | 16.06 | 3.47 | 6.09 |
| YLD12BC1F1(1)-5-128 | 5 | AABBDd | 37.00 | 46.94 | 17.52 | 3.61 | 6.51 |
| YLD12BC1F1(1)-5-130 | 5 | AABbDD | 50.33 | 45.17 | 17.15 | 3.69 | 6.21 |
| YLD12BC1F1(1)-5-137 | 5 | AABBDd | 27.00 | 45.43 | 17.57 | 3.74 | 6.23 |
| YLD12BC1F1(1)-5-152 | 5 | AABBDd | 51.67 | 38.97 | 16.03 | 3.46 | 6.38 |
| YLD12BC1F1(1)-5-156 | 5 | AaBBDD | 44.33 | 34.66 | 14.97 | 3.32 | 6.31 |
| YLD12BC1F1(1)-5-157 | 5 | AABBDd | 50.00 | 39.27 | 15.79 | 3.49 | 6.05 |
| YLD12BC1F1(1)-5-19 | 5 | AABbDD | 33.00 | 37.68 | 15.07 | 3.41 | 5.91 |
| YLD12BC1F1(1)-5-30 | 5 | AABBDd | 47.33 | 39.65 | 15.39 | 3.41 | 5.99 |
| YLD12BC1F1(1)-5-32 | 5 | AABbDD | 42.00 | 33.49 | 13.97 | 3.19 | 5.86 |
| YLD12BC1F1(1)-5-38 | 5 | AABBDd | 40.67 | 39.43 | 15.57 | 3.47 | 5.98 |
| YLD12BC1F1(1)-5-3 | 5 | AaBBDD | 43.00 | 39.46 | 16.26 | 3.49 | 6.37 |
| YLD12BC1F1(1)-5-44 | 5 | AaBBDD | 23.67 | 41.69 | 17.50 | 3.62 | 6.76 |
| YLD12BC1F1(1)-5-62 | 5 | AaBBDD | 29.00 | 36.90 | 15.53 | 3.46 | 6.29 |
| YLD12BC1F1(1)-5-80 | 5 | AABbDD | 43.33 | 39.69 | 16.38 | 3.50 | 6.53 |
| YLD12BC1F1(1)-5-81 | 5 | AABbDD | 49.00 | 37.89 | 15.67 | 3.43 | 6.33 |
| YLD12BC1F1(1)-5-90 | 5 | AaBBDD | 45.67 | 41.68 | 17.02 | 3.57 | 6.74 |
| YLD12BC1F1(1)-5-94 | 5 | AABBDd | 28.00 | 37.86 | 15.37 | 3.38 | 6.03 |
| YLD12BC1F1(1)-5-98 | 5 | AaBBDD | 44.00 | 35.98 | 15.34 | 3.43 | 6.24 |

**Supplementary Table 3.** Phenotypic traits collected for BC_1_F_3_ population derived from 4906-1-6. Traits collected include grain number per head (GNH), thousand grain weight (TGW), grain area (GA), grain width (GW) and grain length (GL). “A”, “B” and “D” designations are given to the wild-type alleles, and “a”, “b” and “d designations are given to the mutant alleles.

| **ID** | **Dosage** | **Genotype** | **GNH** | **TGW (g)** | **GA (mm^2^)** | **GW (mm)** | **GL (mm)** |
| --- | --- | --- | --- | --- | --- | --- | --- |
| YLD12BC1F1(1)_5-23-1 | 0 | aabbdd | 43.00 | 48.99 | 18.10 | 3.86 | 6.27 |
| YLD12BC1F1(1)_5-23-10 | 0 | aabbdd | 50.00 | 39.60 | 16.03 | 3.55 | 6.10 |
| YLD12BC1F1(1)_5-23-2 | 0 | aabbdd | 47.33 | 40.42 | 15.79 | 3.57 | 5.99 |
| YLD12BC1F1(1)_5-23-3 | 0 | aabbdd | 46.00 | 44.78 | 17.01 | 3.75 | 6.05 |
| YLD12BC1F1(1)_5-23-4 | 0 | aabbdd | 43.67 | 38.40 | 15.53 | 3.48 | 6.01 |
| YLD12BC1F1(1)_5-23-5 | 0 | aabbdd | 46.33 | 41.80 | 16.23 | 3.65 | 5.95 |
| YLD12BC1F1(1)_5-23-6 | 0 | aabbdd | 40.67 | 41.48 | 16.13 | 3.61 | 6.01 |
| YLD12BC1F1(1)_5-23-7 | 0 | aabbdd | 44.67 | 42.46 | 16.45 | 3.63 | 6.06 |
| YLD12BC1F1(1)_5-23-8 | 0 | aabbdd | 42.33 | 43.46 | 16.66 | 3.69 | 6.04 |
| YLD12BC1F1(1)_5-23-9 | 0 | aabbdd | 48.33 | 38.41 | 15.49 | 3.51 | 5.94 |
| YLD12BC1F1(1)_5-79-1 | 0 | aabbdd | 36.33 | 40.00 | 15.92 | 3.50 | 6.28 |
| YLD12BC1F1(1)_5-79-11 | 0 | aabbdd | 57.67 | 39.94 | 16.21 | 3.53 | 6.14 |
| YLD12BC1F1(1)_5-79-14 | 0 | aabbdd | 46.67 | 34.93 | 14.80 | 3.37 | 5.98 |
| YLD12BC1F1(1)_5-79-16 | 0 | aabbdd | 49.00 | 35.37 | 14.73 | 3.35 | 5.98 |
| YLD12BC1F1(1)_5-79-20 | 0 | aabbdd | 33.33 | 41.80 | 16.24 | 3.53 | 6.25 |
| YLD12BC1F1(1)_5-79-23 | 0 | aabbdd | 51.33 | 39.61 | 15.84 | 3.48 | 6.14 |
| YLD12BC1F1(1)_5-79-3 | 0 | aabbdd | 47.67 | 38.67 | 15.76 | 3.52 | 6.09 |
| YLD12BC1F1(1)_5-79-4 | 0 | aabbdd | 44.33 | 35.26 | 14.64 | 3.36 | 5.91 |
| YLD12BC1F1(1)_5-96-16 | 0 | aabbdd | 44.00 | 41.74 | 16.63 | 3.59 | 6.37 |
| YLD12BC1F1(1)_5-96-17 | 0 | aabbdd | 53.33 | 43.56 | 17.02 | 3.68 | 6.17 |
| YLD12BC1F1(1)_5-96-2 | 0 | aabbdd | 48.00 | 41.04 | 16.28 | 3.57 | 6.16 |
| YLD12BC1F1(1)_5-96-21 | 0 | aabbdd | 43.67 | 46.95 | 17.72 | 3.74 | 6.38 |
| YLD12BC1F1(1)_5-96-22 | 0 | aabbdd | 52.33 | 42.61 | 16.56 | 3.63 | 6.12 |
| YLD12BC1F1(1)_5-96-27 | 0 | aabbdd | 50.00 | 46.93 | 17.64 | 3.73 | 6.35 |
| YLD12BC1F1(1)_5-96-39 | 0 | aabbdd | 57.33 | 48.14 | 18.19 | 3.80 | 6.47 |
| YLD12BC1F1(1)_5-96-7 | 0 | aabbdd | 53.33 | 42.44 | 16.63 | 3.64 | 6.17 |
| YLD12BC1F1(1)_5-96-8 | 0 | aabbdd | 42.00 | 40.48 | 15.88 | 3.52 | 6.13 |
| YLD12BC1F1(1)_5-79-10 | 1 | Aabbdd | 51.33 | 40.00 | 16.52 | 3.59 | 6.18 |
| YLD12BC1F1(1)_5-79-13 | 1 | Aabbdd | 47.33 | 41.13 | 16.86 | 3.57 | 6.49 |
| YLD12BC1F1(1)_5-79-15 | 1 | Aabbdd | 46.33 | 41.94 | 17.12 | 3.56 | 6.49 |
| YLD12BC1F1(1)_5-79-19 | 1 | Aabbdd | 51.33 | 35.71 | 15.35 | 3.38 | 6.13 |
| YLD12BC1F1(1)_5-79-22 | 1 | Aabbdd | 41.33 | 37.98 | 15.60 | 3.44 | 6.21 |
| YLD12BC1F1(1)_5-79-25 | 1 | Aabbdd | 39.00 | 37.69 | 15.76 | 3.44 | 6.29 |
| YLD12BC1F1(1)_5-79-26 | 1 | Aabbdd | 44.67 | 42.54 | 17.24 | 3.65 | 6.52 |
| YLD12BC1F1(1)_5-79-27 | 1 | Aabbdd | 43.00 | 44.57 | 17.66 | 3.71 | 6.48 |
| YLD12BC1F1(1)_5-79-28 | 1 | Aabbdd | 41.00 | 46.67 | 18.04 | 3.76 | 6.52 |
| YLD12BC1F1(1)_5-79-29 | 1 | Aabbdd | 49.33 | 40.00 | 16.32 | 3.54 | 6.26 |
| YLD12BC1F1(1)_5-79-31 | 1 | Aabbdd | 48.00 | 37.85 | 15.54 | 3.42 | 6.16 |
| YLD12BC1F1(1)_5-79-32 | 1 | Aabbdd | 52.33 | 37.32 | 15.51 | 3.40 | 6.15 |
| YLD12BC1F1(1)_5-79-33 | 1 | Aabbdd | 52.67 | 37.15 | 15.86 | 3.48 | 6.22 |
| YLD12BC1F1(1)_5-79-34 | 1 | Aabbdd | 49.33 | 35.61 | 15.33 | 3.40 | 6.25 |
| YLD12BC1F1(1)_5-79-35 | 1 | Aabbdd | 39.33 | 43.64 | 17.19 | 3.59 | 6.45 |
| YLD12BC1F1(1)_5-79-40 | 1 | Aabbdd | 54.67 | 39.70 | 16.35 | 3.49 | 6.36 |
| YLD12BC1F1(1)_5-79-5 | 1 | Aabbdd | 51.33 | 41.88 | 16.52 | 3.56 | 6.19 |
| YLD12BC1F1(1)_5-79-7 | 1 | Aabbdd | 53.00 | 36.67 | 15.25 | 3.43 | 6.02 |
| YLD12BC1F1(1)_5-79-8 | 1 | Aabbdd | 40.00 | 37.08 | 15.48 | 3.42 | 6.13 |
| YLD12BC1F1(1)_5-96-1 | 1 | Aabbdd | 46.00 | 45.58 | 17.45 | 3.75 | 6.27 |
| YLD12BC1F1(1)_5-96-10 | 1 | Aabbdd | 49.00 | 46.80 | 18.02 | 3.73 | 6.41 |
| YLD12BC1F1(1)_5-96-11 | 1 | Aabbdd | 49.33 | 45.14 | 17.61 | 3.71 | 6.35 |
| YLD12BC1F1(1)_5-96-15 | 1 | Aabbdd | 49.67 | 48.46 | 18.77 | 3.84 | 6.60 |
| YLD12BC1F1(1)_5-96-25 | 1 | Aabbdd | 49.67 | 45.97 | 17.91 | 3.73 | 6.46 |
| YLD12BC1F1(1)_5-96-28 | 1 | Aabbdd | 43.00 | 52.64 | 19.31 | 3.93 | 6.65 |
| YLD12BC1F1(1)_5-96-29 | 1 | Aabbdd | 43.00 | 43.10 | 16.99 | 3.66 | 6.39 |
| YLD12BC1F1(1)_5-96-3 | 1 | Aabbdd | 45.00 | 45.78 | 17.38 | 3.72 | 6.30 |
| YLD12BC1F1(1)_5-96-32 | 1 | Aabbdd | 58.33 | 45.31 | 17.72 | 3.72 | 6.44 |
| YLD12BC1F1(1)_5-96-34 | 1 | Aabbdd | 47.00 | 50.00 | 18.75 | 3.87 | 6.63 |
| YLD12BC1F1(1)_5-96-36 | 1 | Aabbdd | 44.00 | 44.62 | 17.42 | 3.65 | 6.51 |
| YLD12BC1F1(1)_5-96-4 | 1 | Aabbdd | 46.00 | 44.42 | 17.33 | 3.70 | 6.31 |
| YLD12BC1F1(1)_5-96-40 | 1 | Aabbdd | 39.67 | 47.56 | 18.05 | 3.71 | 6.59 |
| YLD12BC1F1(1)_5-96-9 | 1 | Aabbdd | 47.33 | 43.73 | 17.30 | 3.69 | 6.31 |
| YLD12BC1F1(1)_1-2-1 | 2 | aabbDD | 52.33 | 44.01 | 17.15 | 3.68 | 6.24 |
| YLD12BC1F1(1)_1-2-10 | 2 | aabbDD | 51.33 | 49.35 | 18.89 | 3.87 | 6.76 |
| YLD12BC1F1(1)_1-2-2 | 2 | aabbDD | 40.33 | 58.18 | 20.47 | 4.06 | 6.69 |
| YLD12BC1F1(1)_1-2-3 | 2 | aabbDD | 30.33 | 57.25 | 20.27 | 4.03 | 6.76 |
| YLD12BC1F1(1)_1-2-4 | 2 | aabbDD | 39.33 | 54.07 | 19.33 | 3.96 | 6.56 |
| YLD12BC1F1(1)_1-2-5 | 2 | aabbDD | 35.33 | 49.34 | 18.79 | 3.91 | 6.42 |
| YLD12BC1F1(1)_1-2-6 | 2 | aabbDD | 51.00 | 46.80 | 18.09 | 3.74 | 6.52 |
| YLD12BC1F1(1)_1-2-7 | 2 | aabbDD | 35.00 | 55.05 | 19.72 | 3.97 | 6.73 |
| YLD12BC1F1(1)_1-2-9 | 2 | aabbDD | 44.67 | 49.18 | 18.17 | 3.77 | 6.53 |
| YLD12BC1F1(1)_5-27-1 | 2 | aabbDD | 47.33 | 46.13 | 17.90 | 3.79 | 6.35 |
| YLD12BC1F1(1)_5-27-10 | 2 | aabbDD | 52.33 | 44.46 | 17.41 | 3.68 | 6.44 |
| YLD12BC1F1(1)_5-27-2 | 2 | aabbDD | 47.33 | 45.99 | 17.83 | 3.75 | 6.38 |
| YLD12BC1F1(1)_5-27-3 | 2 | aabbDD | 50.00 | 42.33 | 16.95 | 3.67 | 6.22 |
| YLD12BC1F1(1)_5-27-4 | 2 | aabbDD | 50.00 | 45.40 | 18.03 | 3.76 | 6.50 |
| YLD12BC1F1(1)_5-27-5 | 2 | aabbDD | 51.33 | 44.35 | 17.52 | 3.75 | 6.33 |
| YLD12BC1F1(1)_5-27-6 | 2 | aabbDD | 53.67 | 46.09 | 17.59 | 3.77 | 6.37 |
| YLD12BC1F1(1)_5-27-7 | 2 | aabbDD | 49.00 | 42.59 | 17.60 | 3.63 | 6.62 |
| YLD12BC1F1(1)_5-27-8 | 2 | aabbDD | 48.00 | 46.53 | 17.75 | 3.77 | 6.41 |
| YLD12BC1F1(1)_5-27-9 | 2 | aabbDD | 44.00 | 51.67 | 19.14 | 3.87 | 6.72 |
| YLD12BC1F1(1)_1-32-1 | 2 | aaBBdd | 36.67 | 55.00 | 19.32 | 3.97 | 6.55 |
| YLD12BC1F1(1)_1-32-10 | 2 | aaBBdd | 42.33 | 52.44 | 19.04 | 3.90 | 6.67 |
| YLD12BC1F1(1)_1-32-2 | 2 | aaBBdd | 31.33 | 54.26 | 19.11 | 4.01 | 6.43 |
| YLD12BC1F1(1)_1-32-4 | 2 | aaBBdd | 41.67 | 46.16 | 17.51 | 3.72 | 6.35 |
| YLD12BC1F1(1)_1-32-6 | 2 | aaBBdd | 35.33 | 52.45 | 19.03 | 3.86 | 6.63 |
| YLD12BC1F1(1)_1-32-7 | 2 | aaBBdd | 48.00 | 42.43 | 16.82 | 3.65 | 6.19 |
| YLD12BC1F1(1)_1-32-8 | 2 | aaBBdd | 30.33 | 50.00 | 18.15 | 3.84 | 6.31 |
| YLD12BC1F1(1)_1-32-9 | 2 | aaBBdd | 47.67 | 46.64 | 17.67 | 3.77 | 6.36 |
| YLD12BC1F1(1)_5-55-1 | 2 | aaBBdd | 52.00 | 45.32 | 17.58 | 3.76 | 6.24 |
| YLD12BC1F1(1)_5-55-10 | 2 | aaBBdd | 56.00 | 46.31 | 17.59 | 3.72 | 6.39 |
| YLD12BC1F1(1)_5-55-2 | 2 | aaBBdd | 46.00 | 44.71 | 17.75 | 3.70 | 6.50 |
| YLD12BC1F1(1)_5-55-3 | 2 | aaBBdd | 34.33 | 49.51 | 18.58 | 3.75 | 6.67 |
| YLD12BC1F1(1)_5-55-4 | 2 | aaBBdd | 52.33 | 45.22 | 17.52 | 3.74 | 6.32 |
| YLD12BC1F1(1)_5-55-5 | 2 | aaBBdd | 40.00 | 49.50 | 19.00 | 3.82 | 6.73 |
| YLD12BC1F1(1)_5-55-6 | 2 | aaBBdd | 49.67 | 46.11 | 18.42 | 3.76 | 6.74 |
| YLD12BC1F1(1)_5-55-7 | 2 | aaBBdd | 43.67 | 43.44 | 17.59 | 3.69 | 6.55 |
| YLD12BC1F1(1)_5-55-8 | 2 | aaBBdd | 58.00 | 46.44 | 17.62 | 3.77 | 6.31 |
| YLD12BC1F1(1)_5-55-9 | 2 | aaBBdd | 44.00 | 49.02 | 18.68 | 3.89 | 6.51 |
| YLD12BC1F1(1)_5-14-1 | 2 | AAbbdd | 42.00 | 38.49 | 15.72 | 3.41 | 6.27 |
| YLD12BC1F1(1)_5-14-10 | 2 | AAbbdd | 41.33 | 45.16 | 17.20 | 3.67 | 6.36 |
| YLD12BC1F1(1)_5-14-2 | 2 | AAbbdd | 36.33 | 38.53 | 15.88 | 3.47 | 6.21 |
| YLD12BC1F1(1)_5-14-3 | 2 | AAbbdd | 46.67 | 44.36 | 17.35 | 3.63 | 6.46 |
| YLD12BC1F1(1)_5-14-4 | 2 | AAbbdd | 47.00 | 37.80 | 16.02 | 3.44 | 6.34 |
| YLD12BC1F1(1)_5-14-5 | 2 | AAbbdd | 46.33 | 50.14 | 18.80 | 3.84 | 6.57 |
| YLD12BC1F1(1)_5-14-6 | 2 | AAbbdd | 54.33 | 41.35 | 16.48 | 3.60 | 6.22 |
| YLD12BC1F1(1)_5-14-7 | 2 | AAbbdd | 47.33 | 41.83 | 17.45 | 3.62 | 6.53 |
| YLD12BC1F1(1)_5-14-8 | 2 | AAbbdd | 48.33 | 45.86 | 17.88 | 3.76 | 6.43 |
| YLD12BC1F1(1)_5-14-9 | 2 | AAbbdd | 43.00 | 44.50 | 17.10 | 3.63 | 6.40 |
| YLD12BC1F1(1)_5-35-1 | 2 | AAbbdd | 54.00 | 43.46 | 17.14 | 3.68 | 6.22 |
| YLD12BC1F1(1)_5-35-2 | 2 | AAbbdd | 46.00 | 46.59 | 17.59 | 3.70 | 6.52 |
| YLD12BC1F1(1)_5-35-3 | 2 | AAbbdd | 40.00 | 51.92 | 19.28 | 3.93 | 6.65 |
| YLD12BC1F1(1)_5-35-4 | 2 | AAbbdd | 52.33 | 45.41 | 17.78 | 3.77 | 6.30 |
| YLD12BC1F1(1)_5-35-5 | 2 | AAbbdd | 45.67 | 46.72 | 18.10 | 3.79 | 6.41 |
| YLD12BC1F1(1)_5-35-6 | 2 | AAbbdd | 51.67 | 48.00 | 18.20 | 3.77 | 6.49 |
| YLD12BC1F1(1)_5-35-7 | 2 | AAbbdd | 55.00 | 44.06 | 17.38 | 3.72 | 6.23 |
| YLD12BC1F1(1)_5-35-8 | 2 | AAbbdd | 51.00 | 48.17 | 18.17 | 3.76 | 6.45 |
| YLD12BC1F1(1)_5-35-9 | 2 | AAbbdd | 44.33 | 49.10 | 18.64 | 3.83 | 6.61 |
| YLD12BC1F1(1)_5-79-12 | 2 | AAbbdd | 50.67 | 36.51 | 15.78 | 3.43 | 6.22 |
| YLD12BC1F1(1)_5-79-17 | 2 | AAbbdd | 51.67 | 38.00 | 15.74 | 3.44 | 6.25 |
| YLD12BC1F1(1)_5-79-18 | 2 | AAbbdd | 60.00 | 44.78 | 17.38 | 3.69 | 6.37 |
| YLD12BC1F1(1)_5-79-21 | 2 | AAbbdd | 49.67 | 39.80 | 15.95 | 3.48 | 6.18 |
| YLD12BC1F1(1)_5-79-24 | 2 | AAbbdd | 47.00 | 40.35 | 16.25 | 3.49 | 6.35 |
| YLD12BC1F1(1)_5-79-30 | 2 | AAbbdd | 48.00 | 40.00 | 16.37 | 3.47 | 6.45 |
| YLD12BC1F1(1)_5-79-36 | 2 | AAbbdd | 54.00 | 36.98 | 15.76 | 3.47 | 6.13 |
| YLD12BC1F1(1)_5-79-37 | 2 | AAbbdd | 45.67 | 44.82 | 17.59 | 3.68 | 6.52 |
| YLD12BC1F1(1)_5-79-38 | 2 | AAbbdd | 51.00 | 38.76 | 15.99 | 3.41 | 6.38 |
| YLD12BC1F1(1)_5-79-39 | 2 | AAbbdd | 40.00 | 38.33 | 15.53 | 3.42 | 6.29 |
| YLD12BC1F1(1)_5-79-6 | 2 | AAbbdd | 51.00 | 36.67 | 15.37 | 3.39 | 6.12 |
| YLD12BC1F1(1)_5-79-9 | 2 | AAbbdd | 46.33 | 39.86 | 15.97 | 3.49 | 6.23 |
| YLD12BC1F1(1)_5-96-12 | 2 | AAbbdd | 47.67 | 43.01 | 17.05 | 3.62 | 6.40 |
| YLD12BC1F1(1)_5-96-13 | 2 | AAbbdd | 47.67 | 35.10 | 15.32 | 3.34 | 6.27 |
| YLD12BC1F1(1)_5-96-14 | 2 | AAbbdd | 50.00 | 40.53 | 16.68 | 3.56 | 6.35 |
| YLD12BC1F1(1)_5-96-18 | 2 | AAbbdd | 56.67 | 46.24 | 17.58 | 3.70 | 6.39 |
| YLD12BC1F1(1)_5-96-19 | 2 | AAbbdd | 65.00 | 47.44 | 18.32 | 3.82 | 6.50 |
| YLD12BC1F1(1)_5-96-20 | 2 | AAbbdd | 57.00 | 43.86 | 17.22 | 3.63 | 6.39 |
| YLD12BC1F1(1)_5-96-23 | 2 | AAbbdd | 55.67 | 46.53 | 17.94 | 3.76 | 6.42 |
| YLD12BC1F1(1)_5-96-24 | 2 | AAbbdd | 43.33 | 42.85 | 17.00 | 3.61 | 6.41 |
| YLD12BC1F1(1)_5-96-26 | 2 | AAbbdd | 47.67 | 50.63 | 19.03 | 3.84 | 6.71 |
| YLD12BC1F1(1)_5-96-30 | 2 | AAbbdd | 30.67 | 53.15 | 19.71 | 3.84 | 7.01 |
| YLD12BC1F1(1)_5-96-31 | 2 | AAbbdd | 54.00 | 41.36 | 16.39 | 3.52 | 6.27 |
| YLD12BC1F1(1)_5-96-33 | 2 | AAbbdd | 58.00 | 42.13 | 17.13 | 3.65 | 6.35 |
| YLD12BC1F1(1)_5-96-35 | 2 | AAbbdd | 53.33 | 40.56 | 16.73 | 3.57 | 6.30 |
| YLD12BC1F1(1)_5-96-37 | 2 | AAbbdd | 55.33 | 47.65 | 18.30 | 3.76 | 6.59 |
| YLD12BC1F1(1)_5-96-38 | 2 | AAbbdd | 48.00 | 38.13 | 15.85 | 3.45 | 6.33 |
| YLD12BC1F1(1)_5-96-5 | 2 | AAbbdd | 56.00 | 43.63 | 17.33 | 3.64 | 6.46 |
| YLD12BC1F1(1)_5-96-6 | 2 | AAbbdd | 50.00 | 40.20 | 16.27 | 3.52 | 6.27 |
| YLD12BC1F1(1)_5-144-1 | 4 | aaBBDD | 47.67 | 38.32 | 15.84 | 3.45 | 6.17 |
| YLD12BC1F1(1)_5-144-10 | 4 | aaBBDD | 50.00 | 35.93 | 15.28 | 3.34 | 6.32 |
| YLD12BC1F1(1)_5-144-2 | 4 | aaBBDD | 44.67 | 39.03 | 16.08 | 3.44 | 6.30 |
| YLD12BC1F1(1)_5-144-3 | 4 | aaBBDD | 43.33 | 41.31 | 16.72 | 3.52 | 6.43 |
| YLD12BC1F1(1)_5-144-4 | 4 | aaBBDD | 47.67 | 40.77 | 16.53 | 3.53 | 6.41 |
| YLD12BC1F1(1)_5-144-5 | 4 | aaBBDD | 53.00 | 39.75 | 16.46 | 3.54 | 6.27 |
| YLD12BC1F1(1)_5-144-6 | 4 | aaBBDD | 43.00 | 44.42 | 17.47 | 3.65 | 6.58 |
| YLD12BC1F1(1)_5-144-7 | 4 | aaBBDD | 54.67 | 41.95 | 16.95 | 3.59 | 6.42 |
| YLD12BC1F1(1)_5-144-8 | 4 | aaBBDD | 49.00 | 34.35 | 14.84 | 3.27 | 6.16 |
| YLD12BC1F1(1)_5-144-9 | 4 | aaBBDD | 43.67 | 39.69 | 16.34 | 3.51 | 6.37 |
| YLD12BC1F1(1)_5-84-1 | 4 | aaBBDD | 46.67 | 48.21 | 18.07 | 3.85 | 6.33 |
| YLD12BC1F1(1)_5-84-10 | 4 | aaBBDD | 57.33 | 45.58 | 17.68 | 3.72 | 6.39 |
| YLD12BC1F1(1)_5-84-2 | 4 | aaBBDD | 52.67 | 40.82 | 16.42 | 3.57 | 6.20 |
| YLD12BC1F1(1)_5-84-3 | 4 | aaBBDD | 36.00 | 48.70 | 18.16 | 3.75 | 6.55 |
| YLD12BC1F1(1)_5-84-4 | 4 | aaBBDD | 56.33 | 47.28 | 18.26 | 3.76 | 6.53 |
| YLD12BC1F1(1)_5-84-5 | 4 | aaBBDD | 63.33 | 42.79 | 17.00 | 3.61 | 6.31 |
| YLD12BC1F1(1)_5-84-6 | 4 | aaBBDD | 46.00 | 52.46 | 19.33 | 3.94 | 6.69 |
| YLD12BC1F1(1)_5-84-7 | 4 | aaBBDD | 48.33 | 49.38 | 18.64 | 3.79 | 6.62 |
| YLD12BC1F1(1)_5-84-8 | 4 | aaBBDD | 53.33 | 36.31 | 15.37 | 3.38 | 6.16 |
| YLD12BC1F1(1)_5-84-9 | 4 | aaBBDD | 41.33 | 46.21 | 17.95 | 3.72 | 6.48 |
| YLD12BC1F1(1)_1-9-1 | 4 | AAbbDD | 50.67 | 46.12 | 17.96 | 3.69 | 6.48 |
| YLD12BC1F1(1)_1-9-10 | 4 | AAbbDD | 59.67 | 46.76 | 18.05 | 3.75 | 6.50 |
| YLD12BC1F1(1)_1-9-2 | 4 | AAbbDD | 41.33 | 53.23 | 19.57 | 3.91 | 6.70 |
| YLD12BC1F1(1)_1-9-4 | 4 | AAbbDD | 38.67 | 60.86 | 20.65 | 4.10 | 6.79 |
| YLD12BC1F1(1)_1-9-5 | 4 | AAbbDD | 55.33 | 46.93 | 18.14 | 3.75 | 6.47 |
| YLD12BC1F1(1)_1-9-7 | 4 | AAbbDD | 45.00 | 55.70 | 19.70 | 4.00 | 6.67 |
| YLD12BC1F1(1)_1-9-8 | 4 | AAbbDD | 53.33 | 44.94 | 17.16 | 3.71 | 6.15 |
| YLD12BC1F1(1)_5-99-1 | 4 | AAbbDD | 52.33 | 46.62 | 17.94 | 3.73 | 6.47 |
| YLD12BC1F1(1)_5-99-10 | 4 | AAbbDD | 56.67 | 46.29 | 18.05 | 3.70 | 6.57 |
| YLD12BC1F1(1)_5-99-2 | 4 | AAbbDD | 47.67 | 46.36 | 17.92 | 3.76 | 6.40 |
| YLD12BC1F1(1)_5-99-3 | 4 | AAbbDD | 47.00 | 46.67 | 17.91 | 3.72 | 6.49 |
| YLD12BC1F1(1)_5-99-4 | 4 | AAbbDD | 52.00 | 45.83 | 17.67 | 3.72 | 6.37 |
| YLD12BC1F1(1)_5-99-5 | 4 | AAbbDD | 55.00 | 46.48 | 17.82 | 3.74 | 6.34 |
| YLD12BC1F1(1)_5-99-6 | 4 | AAbbDD | 39.33 | 54.41 | 19.54 | 3.91 | 6.78 |
| YLD12BC1F1(1)_5-99-7 | 4 | AAbbDD | 52.33 | 48.66 | 18.42 | 3.78 | 6.51 |
| YLD12BC1F1(1)_5-99-8 | 4 | AAbbDD | 55.33 | 49.94 | 18.31 | 3.83 | 6.41 |
| YLD12BC1F1(1)_5-99-9 | 4 | AAbbDD | 52.67 | 49.43 | 19.05 | 3.82 | 6.88 |
| YLD12BC1F1(1)_5-112-1 | 4 | AABBdd | 46.00 | 41.30 | 16.41 | 3.59 | 6.20 |
| YLD12BC1F1(1)_5-112-10 | 4 | AABBdd | 60.67 | 45.77 | 17.94 | 3.75 | 6.43 |
| YLD12BC1F1(1)_5-112-2 | 4 | AABBdd | 42.00 | 47.22 | 17.87 | 3.74 | 6.37 |
| YLD12BC1F1(1)_5-112-3 | 4 | AABBdd | 46.67 | 49.93 | 18.55 | 3.82 | 6.55 |
| YLD12BC1F1(1)_5-112-4 | 4 | AABBdd | 49.33 | 44.53 | 17.51 | 3.68 | 6.46 |
| YLD12BC1F1(1)_5-112-5 | 4 | AABBdd | 47.67 | 43.64 | 17.15 | 3.71 | 6.21 |
| YLD12BC1F1(1)_5-112-6 | 4 | AABBdd | 43.00 | 47.83 | 17.86 | 3.70 | 6.56 |
| YLD12BC1F1(1)_5-112-7 | 4 | AABBdd | 57.67 | 45.72 | 17.50 | 3.75 | 6.27 |
| YLD12BC1F1(1)_5-112-8 | 4 | AABBdd | 38.00 | 42.19 | 16.43 | 3.61 | 6.26 |
| YLD12BC1F1(1)_5-112-9 | 4 | AABBdd | 44.33 | 36.17 | 15.62 | 3.43 | 6.24 |
| YLD12BC1F1(1)_5-113-1 | 4 | AABBdd | 49.33 | 42.30 | 16.40 | 3.56 | 6.19 |
| YLD12BC1F1(1)_5-113-10 | 4 | AABBdd | 56.33 | 41.12 | 16.62 | 3.58 | 6.29 |
| YLD12BC1F1(1)_5-113-2 | 4 | AABBdd | 47.33 | 46.06 | 17.91 | 3.72 | 6.45 |
| YLD12BC1F1(1)_5-113-3 | 4 | AABBdd | 43.67 | 39.62 | 16.17 | 3.52 | 6.28 |
| YLD12BC1F1(1)_5-113-4 | 4 | AABBdd | 50.00 | 42.20 | 16.67 | 3.59 | 6.31 |
| YLD12BC1F1(1)_5-113-5 | 4 | AABBdd | 52.67 | 42.15 | 16.82 | 3.62 | 6.24 |
| YLD12BC1F1(1)_5-113-6 | 4 | AABBdd | 47.00 | 42.70 | 17.12 | 3.60 | 6.56 |
| YLD12BC1F1(1)_5-113-7 | 4 | AABBdd | 54.00 | 47.22 | 18.19 | 3.83 | 6.42 |
| YLD12BC1F1(1)_5-113-8 | 4 | AABBdd | 52.00 | 46.28 | 17.63 | 3.72 | 6.39 |
| YLD12BC1F1(1)_5-138-1 | 4 | AABBdd | 51.67 | 46.84 | 17.69 | 3.76 | 6.33 |
| YLD12BC1F1(1)_5-138-10 | 4 | AABBdd | 53.67 | 45.16 | 18.09 | 3.69 | 6.71 |
| YLD12BC1F1(1)_5-138-2 | 4 | AABBdd | 49.67 | 45.57 | 17.52 | 3.73 | 6.31 |
| YLD12BC1F1(1)_5-138-3 | 4 | AABBdd | 49.67 | 46.64 | 17.76 | 3.73 | 6.43 |
| YLD12BC1F1(1)_5-138-4 | 4 | AABBdd | 54.33 | 45.03 | 17.68 | 3.66 | 6.56 |
| YLD12BC1F1(1)_5-138-5 | 4 | AABBdd | 45.67 | 47.30 | 18.10 | 3.82 | 6.42 |
| YLD12BC1F1(1)_5-138-6 | 4 | AABBdd | 47.33 | 48.24 | 18.02 | 3.78 | 6.49 |
| YLD12BC1F1(1)_5-138-7 | 4 | AABBdd | 55.67 | 47.72 | 18.17 | 3.79 | 6.46 |
| YLD12BC1F1(1)_5-138-8 | 4 | AABBdd | 46.00 | 47.61 | 18.00 | 3.76 | 6.42 |
| YLD12BC1F1(1)_5-138-9 | 4 | AABBdd | 50.67 | 46.71 | 17.76 | 3.77 | 6.41 |
| YLD12BC1F1(1)_5-147-1 | 4 | AABBdd | 48.33 | 44.14 | 17.32 | 3.61 | 6.49 |
| YLD12BC1F1(1)_5-147-10 | 4 | AABBdd | 59.67 | 41.68 | 17.02 | 3.60 | 6.39 |
| YLD12BC1F1(1)_5-147-2 | 4 | AABBdd | 54.00 | 47.84 | 17.93 | 3.80 | 6.30 |
| YLD12BC1F1(1)_5-147-3 | 4 | AABBdd | 40.00 | 53.25 | 20.26 | 3.91 | 7.06 |
| YLD12BC1F1(1)_5-147-4 | 4 | AABBdd | 48.67 | 49.25 | 18.91 | 3.79 | 6.81 |
| YLD12BC1F1(1)_5-147-5 | 4 | AABBdd | 51.33 | 46.56 | 18.31 | 3.77 | 6.69 |
| YLD12BC1F1(1)_5-147-6 | 4 | AABBdd | 55.67 | 40.84 | 16.75 | 3.56 | 6.45 |
| YLD12BC1F1(1)_5-147-7 | 4 | AABBdd | 41.33 | 40.97 | 16.79 | 3.51 | 6.66 |
| YLD12BC1F1(1)_5-147-8 | 4 | AABBdd | 43.00 | 42.56 | 17.04 | 3.62 | 6.38 |
| YLD12BC1F1(1)_5-147-9 | 4 | AABBdd | 39.33 | 48.73 | 18.80 | 3.78 | 6.79 |
| YLD12BC1F1(1)_1-54-1 | 6 | AABBDD | 45.00 | 43.85 | 17.03 | 3.67 | 6.28 |
| YLD12BC1F1(1)_1-54-10 | 6 | AABBDD | 46.00 | 49.57 | 18.60 | 3.84 | 6.56 |
| YLD12BC1F1(1)_1-54-2 | 6 | AABBDD | 52.33 | 41.02 | 16.74 | 3.61 | 6.28 |
| YLD12BC1F1(1)_1-54-3 | 6 | AABBDD | 43.33 | 45.31 | 17.84 | 3.69 | 6.66 |
| YLD12BC1F1(1)_1-54-4 | 6 | AABBDD | 53.00 | 43.14 | 17.02 | 3.64 | 6.26 |
| YLD12BC1F1(1)_1-54-5 | 6 | AABBDD | 49.00 | 42.24 | 17.14 | 3.62 | 6.47 |
| YLD12BC1F1(1)_1-54-6 | 6 | AABBDD | 44.00 | 46.29 | 17.78 | 3.77 | 6.41 |
| YLD12BC1F1(1)_1-54-7 | 6 | AABBDD | 51.00 | 44.58 | 17.38 | 3.74 | 6.21 |
| YLD12BC1F1(1)_1-54-8 | 6 | AABBDD | 41.33 | 42.34 | 17.30 | 3.60 | 6.51 |
| YLD12BC1F1(1)_1-54-9 | 6 | AABBDD | 53.00 | 42.39 | 16.97 | 3.67 | 6.27 |
| YLD12BC1F1(1)_5-40-1 | 6 | AABBDD | 56.33 | 42.96 | 16.90 | 3.67 | 6.14 |
| YLD12BC1F1(1)_5-40-10 | 6 | AABBDD | 51.33 | 44.74 | 17.23 | 3.69 | 6.28 |
| YLD12BC1F1(1)_5-40-2 | 6 | AABBDD | 51.33 | 44.09 | 17.40 | 3.72 | 6.32 |
| YLD12BC1F1(1)_5-40-3 | 6 | AABBDD | 31.67 | 46.21 | 18.27 | 3.71 | 6.72 |
| YLD12BC1F1(1)_5-40-4 | 6 | AABBDD | 52.67 | 43.29 | 16.95 | 3.63 | 6.29 |
| YLD12BC1F1(1)_5-40-5 | 6 | AABBDD | 50.00 | 41.00 | 16.59 | 3.61 | 6.21 |
| YLD12BC1F1(1)_5-40-6 | 6 | AABBDD | 50.00 | 47.20 | 18.39 | 3.81 | 6.59 |
| YLD12BC1F1(1)_5-40-7 | 6 | AABBDD | 51.00 | 46.80 | 17.83 | 3.81 | 6.28 |
| YLD12BC1F1(1)_5-40-8 | 6 | AABBDD | 40.67 | 42.54 | 16.70 | 3.66 | 6.20 |
| YLD12BC1F1(1)_5-40-9 | 6 | AABBDD | 47.67 | 44.90 | 17.70 | 3.69 | 6.53 |
| YLD12BC1F1(1)_5-9-1 | 6 | AABBDD | 36.33 | 46.61 | 18.17 | 3.81 | 6.43 |
| YLD12BC1F1(1)_5-9-10 | 6 | AABBDD | 34.67 | 46.25 | 18.12 | 3.78 | 6.52 |
| YLD12BC1F1(1)_5-9-2 | 6 | AABBDD | 34.67 | 44.33 | 17.06 | 3.70 | 6.22 |
| YLD12BC1F1(1)_5-9-4 | 6 | AABBDD | 50.33 | 44.50 | 17.59 | 3.73 | 6.40 |
| YLD12BC1F1(1)_5-9-5 | 6 | AABBDD | 56.67 | 45.94 | 18.13 | 3.76 | 6.61 |
| YLD12BC1F1(1)_5-9-6 | 6 | AABBDD | 33.67 | 49.11 | 18.73 | 3.82 | 6.68 |
| YLD12BC1F1(1)_5-9-7 | 6 | AABBDD | 39.00 | 47.18 | 18.14 | 3.80 | 6.49 |
| YLD12BC1F1(1)_5-9-8 | 6 | AABBDD | 30.33 | 46.37 | 18.12 | 3.77 | 6.51 |
| YLD12BC1F1(1)_5-9-9 | 6 | AABBDD | 39.33 | 45.25 | 17.93 | 3.71 | 6.58 |

**Supplementary Table 4.** Genotypes of lines derived from the C538-1 line. Genotyping was conducted by NGS of pooled PCR amplicons. “A”, “B” and “D” designations are given to the wild-type alleles, and “a”, “b” and “d designations are given to the mutant alleles.

| **Line** | **TaGS3 genotype based on NGS of PCR amplicons** |
| --- | --- |
| C538-1-78-2-24 | aabbDD |
| C538-1-78-2-17 | aaBbDD |
| C538-1-78-2-19 | aaBbDD |
| C538-1-78-2-53 | aaBbDD |
| C538-1-78-2-58 | aaBbDD |
| C538-1-78-2-41 | aaBBDD |
| C538-1-78-2-48 | aaBBDD |
| C538-1-78-2-33 | AabbDD |
| C538-1-78-2-18 | AabbDD |
| C538-1-78-2-14 | AabbDD |
| C538-1-78-2-2 | AabbDD |
| C538-1-78-2-3 | AabbDD |
| C538-1-78-2-4 | AabbDD |
| C538-1-78-2-6 | AabbDD |
| C538-1-78-2-8 | AabbDD |
| C538-1-78-2-20 | AabbDD |
| C538-1-78-2-35 | AabbDD |
| C538-1-78-2-37 | AabbDD |
| C538-1-78-2-40 | AabbDD |
| C538-1-78-2-46 | AabbDD |
| C538-1-78-2-47 | AabbDD |
| C538-1-78-2-55 | AabbDD |
| C538-1-78-2-60 | AabbDD |
| C538-1-78-2-22 | AaBbDd |
| C538-1-78-2-21 | AaBbDD |
| C538-1-78-2-50 | AaBbDD |
| C538-1-78-2-10 | AaBbDD |
| C538-1-78-2-1 | AaBbDD |
| C538-1-78-2-5 | AaBbDD |
| C538-1-78-2-11 | AaBbDD |
| C538-1-78-2-23 | AaBbDD |
| C538-1-78-2-28 | AaBbDD |
| C538-1-78-2-29 | AaBbDD |
| C538-1-78-2-31 | AaBbDD |
| C538-1-78-2-38 | AaBbDD |
| C538-1-78-2-49 | AaBbDD |
| C538-1-78-2-52 | AaBBDD |
| C538-1-90-10-18 | AaBBDD |
| C538-1-78-2-42 | AaBBDD |
| C538-1-19-7-1 | AaBBDD |
| C538-1-78-2-7 | AaBBDD |
| C538-1-78-2-15 | AaBBDD |
| C538-1-78-2-25 | AaBBDD |
| C538-1-90-10-1 | AaBBDD |
| C538-1-90-10-14 | AaBBDD |
| C538-1-66-19-1 | AAbbdd |
| C538-1-66-19-21 | AAbbdd |
| C538-1-66-19-15 | AAbbdd |
| C538-1-66-19-16 | AAbbdd |
| C538-1-66-19-7 | AAbbdd |
| C538-1-66-19-14 | AAbbdd |
| C538-1-66-19-10 | AAbbdd |
| C538-1-66-9-22 | AAbbdd |
| C538-1-66-9-27 | AAbbdd |
| C538-1-66-19-2 | AAbbdd |
| C538-1-66-19-3 | AAbbdd |
| C538-1-66-19-4 | AAbbdd |
| C538-1-66-19-5 | AAbbdd |
| C538-1-66-19-6 | AAbbdd |
| C538-1-66-19-8 | AAbbdd |
| C538-1-66-19-9 | AAbbdd |
| C538-1-66-19-11 | AAbbdd |
| C538-1-66-19-12 | AAbbdd |
| C538-1-66-19-13 | AAbbdd |
| C538-1-66-19-17 | AAbbdd |
| C538-1-66-19-18 | AAbbdd |
| C538-1-66-19-19 | AAbbdd |
| C538-1-66-19-20 | AAbbdd |
| C538-1-66-19-22 | AAbbdd |
| C538-1-66-19-23 | AAbbdd |
| C538-1-66-19-24 | AAbbdd |
| C538-1-66-19-25 | AAbbdd |
| C538-1-66-19-26 | AAbbdd |
| C538-1-66-19-27 | AAbbdd |
| C538-1-66-19-28 | AAbbdd |
| C538-1-66-19-29 | AAbbdd |
| C538-1-66-19-30 | AAbbdd |
| C538-1-66-9-2 | AAbbdd |
| C538-1-66-9-4 | AAbbdd |
| C538-1-66-9-10 | AAbbdd |
| C538-1-66-9-17 | AAbbdd |
| C538-1-66-9-30 | AAbbdd |
| C538-1-66-9-11 | AAbbDd |
| C538-1-66-9-1 | AAbbDd |
| C538-1-66-9-3 | AAbbDd |
| C538-1-66-9-5 | AAbbDd |
| C538-1-66-9-6 | AAbbDd |
| C538-1-66-9-9 | AAbbDd |
| C538-1-66-9-15 | AAbbDd |
| C538-1-66-9-16 | AAbbDd |
| C538-1-66-9-18 | AAbbDd |
| C538-1-66-9-19 | AAbbDd |
| C538-1-66-9-20 | AAbbDd |
| C538-1-66-9-28 | AAbbDd |
| C538-1-66-9-29 | AAbbDd |
| C538-1-78-2-13 | AAbbDD |
| C538-1-61-1-3 | AAbbDD |
| C538-1-61-1-19 | AAbbDD |
| C538-1-61-1-18 | AAbbDD |
| C538-1-61-1-11 | AAbbDD |
| C538-1-61-1-14 | AAbbDD |
| C538-1-66-9-13 | AAbbDD |
| C538-1-66-9-25 | AAbbDD |
| C538-1-66-9-23 | AAbbDD |
| C538-1-66-9-8 | AAbbDD |
| C538-1-66-9-14 | AAbbDD |
| C538-1-66-9-21 | AAbbDD |
| C538-1-66-9-24 | AAbbDD |
| C538-1-66-9-26 | AAbbDD |
| C538-1-78-2-30 | AAbbDD |
| C538-1-78-2-34 | AAbbDD |
| C538-1-78-2-43 | AAbbDD |
| C538-1-78-2-44 | AAbbDD |
| C538-1-78-2-54 | AAbbDD |
| C538-1-61-1-4 | AAbbDD |
| C538-1-61-1-5 | AAbbDD |
| C538-1-61-1-7 | AAbbDD |
| C538-1-61-1-13 | AAbbDD |
| C538-1-61-1-21 | AAbbDD |
| C538-1-61-1-26 | AAbbDD |
| C538-1-78-2-56 | AABbDd |
| C538-1-78-2-39 | AABbDd |
| C538-1-78-2-36 | AABbDD |
| C538-1-19-10-20 | AABbDD |
| C538-1-19-10-30 | AABbDD |
| C538-1-19-6-2 | AABbDD |
| C538-1-66-5-2 | AABbDD |
| C538-1-78-2-9 | AABbDD |
| C538-1-78-2-45 | AABbDD |
| C538-1-78-2-57 | AABbDD |
| C538-1-78-13-4 | AABbDD |
| C538-1-90-10-3 | AABbDD |
| C538-1-90-10-20 | AABbDD |
| C538-1-61-1-2 | AABbDD |
| C538-1-61-1-9 | AABbDD |
| C538-1-61-1-10 | AABbDD |
| C538-1-61-1-15 | AABbDD |
| C538-1-61-1-17 | AABbDD |
| C538-1-61-1-20 | AABbDD |
| C538-1-61-1-23 | AABbDD |
| C538-1-61-1-29 | AABbDD |
| C538-1-61-1-30 | AABbDD |
| C538-1-92-10-10 | AABBdd |
| C538-1-53-7-2 | AABBdd |
| C538-1-92-10-5 | AABBdd |
| C538-1-53-10-5 | AABBdd |
| C538-1-19-7-4 | AABBdd |
| C538-1-53-7-1 | AABBdd |
| C538-1-53-7-3 | AABBdd |
| C538-1-53-7-4 | AABBdd |
| C538-1-53-7-5 | AABBdd |
| C538-1-53-10-1 | AABBdd |
| C538-1-53-10-2 | AABBdd |
| C538-1-53-10-3 | AABBdd |
| C538-1-53-10-4 | AABBdd |
| C538-1-92-6-1 | AABBdd |
| C538-1-92-6-2 | AABBdd |
| C538-1-92-6-3 | AABBdd |
| C538-1-92-6-4 | AABBdd |
| C538-1-92-6-5 | AABBdd |
| C538-1-92-8-1 | AABBdd |
| C538-1-92-8-2 | AABBdd |
| C538-1-92-8-3 | AABBdd |
| C538-1-92-8-4 | AABBdd |
| C538-1-92-8-5 | AABBdd |
| C538-1-92-10-1 | AABBdd |
| C538-1-92-10-2 | AABBdd |
| C538-1-92-10-3 | AABBdd |
| C538-1-92-10-4 | AABBdd |
| C538-1-92-10-6 | AABBdd |
| C538-1-92-10-8 | AABBdd |
| C538-1-92-10-9 | AABBdd |
| C538-1-19-10-26 | AABBDd |
| C538-1-19-10-27 | AABBDd |
| C538-1-78-14-1 | AABBDd |
| C538-1-90-10-27 | AABBDd |
| C538-1-78-2-59 | AABBDD |
| C538-1-90-10-19 | AABBDD |
| C538-1-19-10-14 | AABBDD |
| C538-1-19-6-1 | AABBDD |
| C538-1-78-13-5 | AABBDD |
| C538-1-90-10-21 | AABBDD |
| C538-1-19-10-15 | AABBDD |
| C538-1-90-10-29 | AABBDD |
| C538-1-19-10-8 | AABBDD |
| C538-1-90-10-5 | AABBDD |
| C538-1-66-14-3 | AABBDD |
| C538-1-78-20-1 | AABBDD |
| C538-1-92-10-7 | AABBDD |
| C538-1-19-10-23 | AABBDD |
| C538-1-19-10-12 | AABBDD |
| C538-1-90-10-22 | AABBDD |
| C538-1-78-13-3 | AABBDD |
| C538-1-19-10-1 | AABBDD |
| C538-1-19-10-2 | AABBDD |
| C538-1-19-10-3 | AABBDD |
| C538-1-19-10-4 | AABBDD |
| C538-1-19-10-5 | AABBDD |
| C538-1-19-10-6 | AABBDD |
| C538-1-19-10-7 | AABBDD |
| C538-1-19-10-9 | AABBDD |
| C538-1-19-10-10 | AABBDD |
| C538-1-19-10-11 | AABBDD |
| C538-1-19-10-13 | AABBDD |
| C538-1-19-10-16 | AABBDD |
| C538-1-19-10-17 | AABBDD |
| C538-1-19-10-18 | AABBDD |
| C538-1-19-10-19 | AABBDD |
| C538-1-19-10-21 | AABBDD |
| C538-1-19-10-24 | AABBDD |
| C538-1-19-10-25 | AABBDD |
| C538-1-19-10-28 | AABBDD |
| C538-1-19-10-29 | AABBDD |
| C538-1-19-6-3 | AABBDD |
| C538-1-19-6-4 | AABBDD |
| C538-1-19-6-5 | AABBDD |
| C538-1-19-7-2 | AABBDD |
| C538-1-19-7-3 | AABBDD |
| C538-1-19-7-5 | AABBDD |
| C538-1-66-5-1 | AABBDD |
| C538-1-66-5-3 | AABBDD |
| C538-1-66-5-4 | AABBDD |
| C538-1-66-5-5 | AABBDD |
| C538-1-66-14-1 | AABBDD |
| C538-1-66-14-2 | AABBDD |
| C538-1-66-14-4 | AABBDD |
| C538-1-66-14-5 | AABBDD |
| C538-1-78-2-12 | AABBDD |
| C538-1-78-2-27 | AABBDD |
| C538-1-78-20-2 | AABBDD |
| C538-1-78-20-3 | AABBDD |
| C538-1-78-20-5 | AABBDD |
| C538-1-78-20-6 | AABBDD |
| C538-1-78-20-7 | AABBDD |
| C538-1-78-20-8 | AABBDD |
| C538-1-78-20-9 | AABBDD |
| C538-1-78-20-10 | AABBDD |
| C538-1-78-13-2 | AABBDD |
| C538-1-78-14-2 | AABBDD |
| C538-1-78-14-3 | AABBDD |
| C538-1-78-14-4 | AABBDD |
| C538-1-78-14-5 | AABBDD |
| C538-1-53-4-1 | AABBDD |
| C538-1-53-4-2 | AABBDD |
| C538-1-53-4-3 | AABBDD |
| C538-1-53-6-1 | AABBDD |
| C538-1-53-6-2 | AABBDD |
| C538-1-53-6-3 | AABBDD |
| C538-1-53-6-4 | AABBDD |
| C538-1-53-6-5 | AABBDD |
| C538-1-90-10-2 | AABBDD |
| C538-1-90-10-4 | AABBDD |
| C538-1-90-10-6 | AABBDD |
| C538-1-90-10-7 | AABBDD |
| C538-1-90-10-8 | AABBDD |
| C538-1-90-10-9 | AABBDD |
| C538-1-90-10-10 | AABBDD |
| C538-1-90-10-11 | AABBDD |
| C538-1-90-10-12 | AABBDD |
| C538-1-90-10-13 | AABBDD |
| C538-1-90-10-15 | AABBDD |
| C538-1-90-10-16 | AABBDD |
| C538-1-90-10-17 | AABBDD |
| C538-1-90-10-23 | AABBDD |
| C538-1-90-10-24 | AABBDD |
| C538-1-90-10-25 | AABBDD |
| C538-1-90-10-26 | AABBDD |
| C538-1-61-1-1 | AABBDD |
| C538-1-61-1-6 | AABBDD |
| C538-1-61-1-8 | AABBDD |
| C538-1-61-1-12 | AABBDD |
| C538-1-61-1-16 | AABBDD |
| C538-1-61-1-22 | AABBDD |
| C538-1-61-1-24 | AABBDD |
| C538-1-61-1-25 | AABBDD |
| C538-1-61-1-27 | AABBDD |
| C538-1-61-1-28 | AABBDD |

**Supplementary Table 5.** Phenotypic traits collected for T_5_ generation population derived from C538-1. Traits collected include grain number per head (GNH), thousand grain weight (TGW), grain area (GA), grain width (GW) and grain length (GL). “A”, “B” and “D” designations are given to the wild-type alleles, and “a”, “b” and “d designations are given to the mutant alleles.

| **ID** | **Dosage** | **Genotype** | **GNH** | **TGW (g)** | **GA (mm^2^)** | **GW (mm)** | **GL (mm)** |
| --- | --- | --- | --- | --- | --- | --- | --- |
| C538-1-78-2-22-11-11 | 0 | aabbdd | 32.33 | 37.53 | 14.59 | 3.43 | 5.74 |
| C538-1-78-2-22-11-20 | 0 | aabbdd | 40.00 | 40.17 | 15.13 | 3.55 | 5.66 |
| C538-1-78-2-22-11-22 | 0 | aabbdd | 36.00 | 40.65 | 15.27 | 3.59 | 5.67 |
| C538-1-78-2-22-11-26 | 0 | aabbdd | 51.67 | 41.35 | 15.70 | 3.62 | 5.75 |
| C538-1-78-2-22-16-1 | 0 | aabbdd | 44.00 | 40.15 | 15.25 | 3.55 | 5.69 |
| C538-1-78-2-22-16-10 | 0 | aabbdd | 33.00 | 39.80 | 15.49 | 3.51 | 5.83 |
| C538-1-78-2-22-16-2 | 0 | aabbdd | 38.33 | 40.35 | 15.44 | 3.61 | 5.72 |
| C538-1-78-2-22-16-3 | 0 | aabbdd | 50.67 | 39.28 | 15.22 | 3.53 | 5.75 |
| C538-1-78-2-22-16-4 | 0 | aabbdd | 42.33 | 38.58 | 15.12 | 3.51 | 5.76 |
| C538-1-78-2-22-16-5 | 0 | aabbdd | 58.33 | 40.46 | 15.52 | 3.61 | 5.70 |
| C538-1-78-2-22-16-6 | 0 | aabbdd | 52.67 | 39.43 | 15.24 | 3.57 | 5.66 |
| C538-1-78-2-22-16-7 | 0 | aabbdd | 47.00 | 41.21 | 15.46 | 3.64 | 5.68 |
| C538-1-78-2-22-16-8 | 0 | aabbdd | 39.00 | 37.35 | 14.81 | 3.46 | 5.67 |
| C538-1-78-2-22-16-9 | 0 | aabbdd | 41.00 | 38.37 | 15.08 | 3.51 | 5.69 |
| C538-1-78-2-22-27-1 | 0 | aabbdd | 51.33 | 39.42 | 15.08 | 3.57 | 5.64 |
| C538-1-78-2-22-27-10 | 0 | aabbdd | 42.67 | 39.77 | 15.21 | 3.51 | 5.75 |
| C538-1-78-2-22-27-2 | 0 | aabbdd | 49.00 | 40.88 | 15.59 | 3.65 | 5.73 |
| C538-1-78-2-22-27-3 | 0 | aabbdd | 26.67 | 41.75 | 15.81 | 3.61 | 5.88 |
| C538-1-78-2-22-27-4 | 0 | aabbdd | 35.00 | 37.33 | 14.68 | 3.48 | 5.66 |
| C538-1-78-2-22-27-5 | 0 | aabbdd | 50.67 | 41.12 | 15.39 | 3.62 | 5.64 |
| C538-1-78-2-22-27-7 | 0 | aabbdd | 43.67 | 40.53 | 15.22 | 3.62 | 5.61 |
| C538-1-78-2-22-27-8 | 0 | aabbdd | 24.00 | 40.97 | 15.47 | 3.51 | 5.88 |
| C538-1-78-2-22-27-9 | 0 | aabbdd | 42.00 | 40.48 | 15.22 | 3.59 | 5.60 |
| C538-1-78-2-22-37-1 | 0 | aabbdd | 45.00 | 38.22 | 14.70 | 3.50 | 5.62 |
| C538-1-78-2-22-37-2 | 0 | aabbdd | 37.67 | 37.70 | 14.66 | 3.48 | 5.58 |
| C538-1-78-2-22-37-4 | 0 | aabbdd | 37.00 | 41.35 | 15.77 | 3.61 | 5.78 |
| C538-1-78-2-22-37-5 | 0 | aabbdd | 49.67 | 38.93 | 15.07 | 3.53 | 5.65 |
| C538-1-78-2-22-37-6 | 0 | aabbdd | 43.67 | 34.50 | 14.25 | 3.40 | 5.58 |
| C538-1-78-2-22-37-8 | 0 | aabbdd | 28.33 | 38.47 | 14.92 | 3.55 | 5.58 |
| C538-1-78-2-22-37-9 | 0 | aabbdd | 47.33 | 41.27 | 15.74 | 3.59 | 5.85 |
| C538-1-78-2-22-41-13 | 0 | aabbdd | 27.00 | 37.78 | 14.32 | 3.49 | 5.49 |
| C538-1-78-2-22-41-15 | 0 | aabbdd | 24.33 | 38.22 | 14.89 | 3.48 | 5.70 |
| C538-1-78-2-22-46-13 | 0 | aabbdd | 41.00 | 38.70 | 15.03 | 3.51 | 5.73 |
| C538-1-78-2-22-46-16 | 0 | aabbdd | 55.67 | 39.16 | 15.01 | 3.51 | 5.95 |
| C538-1-78-2-22-46-22 | 0 | aabbdd | 44.33 | 40.15 | 15.28 | 3.53 | 5.73 |
| C538-1-78-2-22-46-27 | 0 | aabbdd | 47.67 | 39.02 | 14.97 | 3.54 | 5.63 |
| C538-1-78-2-22-46-4 | 0 | aabbdd | 40.67 | 39.84 | 15.04 | 3.54 | 5.70 |
| C538-1-78-2-22-47-10 | 0 | aabbdd | 42.67 | 40.78 | 15.23 | 3.52 | 5.74 |
| C538-1-78-2-22-47-11 | 0 | aabbdd | 47.33 | 40.63 | 15.36 | 3.58 | 5.86 |
| C538-1-78-2-22-47-14 | 0 | aabbdd | 55.00 | 38.91 | 14.99 | 3.52 | 5.68 |
| C538-1-78-2-22-47-17 | 0 | aabbdd | 44.33 | 38.20 | 15.15 | 3.54 | 5.77 |
| C538-1-78-2-22-47-20 | 0 | aabbdd | 31.00 | 38.60 | 15.09 | 3.52 | 5.69 |
| C538-1-78-2-22-47-24 | 0 | aabbdd | 39.00 | 39.74 | 14.95 | 3.51 | 5.60 |
| C538-1-78-2-22-47-25 | 0 | aabbdd | 44.67 | 41.42 | 15.49 | 3.60 | 5.69 |
| C538-1-78-2-22-47-29 | 0 | aabbdd | 44.33 | 40.90 | 15.63 | 3.58 | 5.79 |
| C538-1-78-2-22-47-6 | 0 | aabbdd | 35.00 | 38.19 | 14.42 | 3.49 | 5.52 |
| C538-1-78-2-22-76-2 | 0 | aabbdd | 47.33 | 40.77 | 15.47 | 3.61 | 5.72 |
| C538-1-78-2-22-76-3 | 0 | aabbdd | 50.33 | 37.09 | 14.97 | 3.44 | 5.75 |
| C538-1-78-2-22-76-7 | 0 | aabbdd | 32.00 | 43.75 | 16.22 | 3.77 | 5.68 |
| C538-1-78-2-22-76-8 | 0 | aabbdd | 29.33 | 43.98 | 16.30 | 3.70 | 5.83 |
| C538-1-78-2-22-76-9 | 0 | aabbdd | 30.67 | 41.52 | 16.20 | 3.71 | 5.81 |
| C538-1-78-2-22-41-10 | 1 | aabbDd | 45.67 | 41.31 | 15.71 | 3.57 | 5.85 |
| C538-1-78-2-22-41-12 | 1 | aabbDd | 51.00 | 39.80 | 15.42 | 3.54 | 5.82 |
| C538-1-78-2-22-41-3 | 1 | aabbDd | 45.33 | 41.32 | 15.71 | 3.59 | 5.81 |
| C538-1-78-2-22-41-4 | 1 | aabbDd | 46.00 | 41.45 | 15.69 | 3.54 | 5.88 |
| C538-1-78-2-22-41-6 | 1 | aabbDd | 24.00 | 38.75 | 15.05 | 3.48 | 5.85 |
| C538-1-78-2-22-41-8 | 1 | aabbDd | 35.00 | 38.57 | 15.06 | 3.43 | 5.85 |
| C538-1-78-2-22-11-1 | 1 | aaBbdd | 52.33 | 40.51 | 15.53 | 3.55 | 5.84 |
| C538-1-78-2-22-11-12 | 1 | aaBbdd | 57.00 | 34.91 | 14.52 | 3.30 | 5.97 |
| C538-1-78-2-22-11-14 | 1 | aaBbdd | 50.33 | 40.60 | 15.54 | 3.56 | 5.78 |
| C538-1-78-2-22-11-17 | 1 | aaBbdd | 52.67 | 37.28 | 14.74 | 3.44 | 5.77 |
| C538-1-78-2-22-11-2 | 1 | aaBbdd | 43.67 | 39.92 | 15.46 | 3.47 | 5.94 |
| C538-1-78-2-22-11-23 | 1 | aaBbdd | 35.33 | 40.38 | 15.38 | 3.59 | 5.73 |
| C538-1-78-2-22-11-24 | 1 | aaBbdd | 41.00 | 39.84 | 15.40 | 3.52 | 5.82 |
| C538-1-78-2-22-11-25 | 1 | aaBbdd | 44.00 | 40.53 | 15.55 | 3.56 | 5.77 |
| C538-1-78-2-22-11-27 | 1 | aaBbdd | 45.67 | 42.92 | 16.09 | 3.63 | 5.86 |
| C538-1-78-2-22-11-28 | 1 | aaBbdd | 41.00 | 37.56 | 15.05 | 3.47 | 5.80 |
| C538-1-78-2-22-11-3 | 1 | aaBbdd | 37.00 | 36.85 | 14.83 | 3.41 | 5.82 |
| C538-1-78-2-22-11-30 | 1 | aaBbdd | 32.33 | 39.38 | 15.53 | 3.56 | 5.88 |
| C538-1-78-2-22-11-4 | 1 | aaBbdd | 49.67 | 39.87 | 15.52 | 3.55 | 5.80 |
| C538-1-78-2-22-11-5 | 1 | aaBbdd | 45.00 | 41.19 | 15.75 | 3.60 | 5.92 |
| C538-1-78-2-22-11-6 | 1 | aaBbdd | 35.33 | 41.32 | 15.57 | 3.57 | 5.77 |
| C538-1-78-2-22-11-7 | 1 | aaBbdd | 31.67 | 41.05 | 15.74 | 3.53 | 5.96 |
| C538-1-78-2-22-11-8 | 1 | aaBbdd | 36.33 | 38.26 | 15.10 | 3.45 | 5.86 |
| C538-1-78-2-22-30-20 | 1 | aaBbdd | 40.33 | 39.67 | 15.27 | 3.50 | 5.81 |
| C538-1-78-2-22-30-24 | 1 | aaBbdd | 35.67 | 40.19 | 15.29 | 3.55 | 5.77 |
| C538-1-78-2-22-30-34 | 1 | aaBbdd | 39.00 | 39.40 | 15.39 | 3.53 | 5.81 |
| C538-1-78-2-22-30-36 | 1 | aaBbdd | 35.00 | 42.67 | 16.15 | 3.62 | 5.96 |
| C538-1-78-2-22-30-55 | 1 | aaBbdd | 44.33 | 43.01 | 15.95 | 3.63 | 5.89 |
| C538-1-78-2-22-30-9 | 1 | aaBbdd | 49.67 | 41.28 | 15.68 | 3.56 | 5.88 |
| C538-1-78-2-22-46-1 | 1 | Aabbdd | 51.00 | 41.76 | 16.01 | 3.58 | 5.97 |
| C538-1-78-2-22-46-11 | 1 | Aabbdd | 37.00 | 40.18 | 15.43 | 3.49 | 5.86 |
| C538-1-78-2-22-46-2 | 1 | Aabbdd | 52.00 | 40.77 | 15.69 | 3.54 | 5.90 |
| C538-1-78-2-22-46-20 | 1 | Aabbdd | 51.00 | 40.98 | 15.72 | 3.56 | 5.85 |
| C538-1-78-2-22-46-21 | 1 | Aabbdd | 41.00 | 40.98 | 15.57 | 3.57 | 5.80 |
| C538-1-78-2-22-46-24 | 1 | Aabbdd | 37.67 | 38.94 | 15.18 | 3.49 | 5.81 |
| C538-1-78-2-22-46-28 | 1 | Aabbdd | 41.00 | 40.89 | 16.10 | 3.53 | 6.06 |
| C538-1-78-2-22-46-29 | 1 | Aabbdd | 45.33 | 40.44 | 15.95 | 3.53 | 6.00 |
| C538-1-78-2-22-46-30 | 1 | Aabbdd | 56.33 | 41.24 | 16.21 | 3.57 | 6.04 |
| C538-1-78-2-22-46-8 | 1 | Aabbdd | 36.67 | 35.82 | 14.62 | 3.35 | 5.85 |
| C538-1-78-2-22-47-1 | 1 | Aabbdd | 58.67 | 39.49 | 15.56 | 3.53 | 5.86 |
| C538-1-78-2-22-47-12 | 1 | Aabbdd | 42.67 | 41.48 | 15.73 | 3.60 | 5.89 |
| C538-1-78-2-22-47-13 | 1 | Aabbdd | 46.33 | 38.56 | 14.93 | 3.46 | 5.75 |
| C538-1-78-2-22-47-16 | 1 | Aabbdd | 46.33 | 39.78 | 15.15 | 3.50 | 5.87 |
| C538-1-78-2-22-47-18 | 1 | Aabbdd | 46.67 | 38.93 | 15.12 | 3.47 | 5.82 |
| C538-1-78-2-22-47-19 | 1 | Aabbdd | 42.33 | 39.29 | 15.24 | 3.53 | 5.74 |
| C538-1-78-2-22-47-2 | 1 | Aabbdd | 52.33 | 40.57 | 15.56 | 3.56 | 5.82 |
| C538-1-78-2-22-47-22 | 1 | Aabbdd | 51.33 | 41.36 | 15.62 | 3.57 | 5.82 |
| C538-1-78-2-22-47-27 | 1 | Aabbdd | 40.33 | 41.16 | 15.78 | 3.58 | 5.86 |
| C538-1-78-2-22-47-3 | 1 | Aabbdd | 52.33 | 39.81 | 15.32 | 3.51 | 5.81 |
| C538-1-78-2-22-47-4 | 1 | Aabbdd | 33.67 | 41.19 | 15.47 | 3.61 | 5.73 |
| C538-1-78-2-22-47-8 | 1 | Aabbdd | 43.00 | 39.15 | 15.51 | 3.48 | 5.93 |
| C538-1-78-2-22-47-9 | 1 | Aabbdd | 50.00 | 38.73 | 15.16 | 3.49 | 5.80 |
| C538-1-78-2-22-41-1 | 2 | aabbDD | 30.67 | 39.35 | 15.15 | 3.47 | 5.84 |
| C538-1-78-2-22-41-11 | 2 | aabbDD | 39.00 | 40.51 | 15.62 | 3.53 | 5.89 |
| C538-1-78-2-22-41-7 | 2 | aabbDD | 32.67 | 39.29 | 15.25 | 3.51 | 5.83 |
| C538-1-78-2-22-14-29 | 2 | aaBbDd | 30.33 | 37.47 | 14.67 | 3.35 | 6.00 |
| C538-1-78-2-22-30-25 | 2 | aaBbDd | 37.67 | 40.27 | 15.32 | 3.52 | 5.77 |
| C538-1-78-2-22-30-54 | 2 | aaBbDd | 60.67 | 41.70 | 15.94 | 3.60 | 5.88 |
| C538-1-78-2-22-11-16 | 2 | aaBBdd | 46.67 | 36.64 | 14.55 | 3.42 | 5.64 |
| C538-1-78-2-22-14-2 | 2 | aaBBdd | 39.33 | 36.10 | 14.22 | 3.30 | 5.77 |
| C538-1-78-2-22-14-23 | 2 | aaBBdd | 30.00 | 41.22 | 15.25 | 3.54 | 5.77 |
| C538-1-78-2-22-14-24 | 2 | aaBBdd | 50.00 | 38.20 | 15.02 | 3.47 | 5.72 |
| C538-1-78-2-22-14-6 | 2 | aaBBdd | 44.33 | 40.23 | 15.27 | 3.54 | 5.74 |
| C538-1-78-2-22-30-18 | 2 | AabbDd | 46.33 | 40.14 | 15.46 | 3.52 | 5.85 |
| C538-1-78-2-22-30-28 | 2 | AabbDd | 40.33 | 39.01 | 15.24 | 3.47 | 5.85 |
| C538-1-78-2-22-30-33 | 2 | AabbDd | 34.00 | 39.90 | 15.36 | 3.54 | 5.82 |
| C538-1-78-2-22-30-39 | 2 | AabbDd | 21.67 | 43.85 | 16.22 | 3.60 | 6.04 |
| C538-1-78-2-22-30-50 | 2 | AabbDd | 41.33 | 40.89 | 15.63 | 3.56 | 5.86 |
| C538-1-78-2-22-30-68 | 2 | AabbDd | 67.00 | 36.02 | 14.44 | 3.38 | 5.63 |
| C538-1-78-2-22-30-8 | 2 | AabbDd | 52.33 | 42.29 | 16.14 | 3.60 | 5.98 |
| C538-1-78-2-22-30-10 | 2 | AaBbdd | 52.67 | 43.04 | 16.03 | 3.61 | 5.92 |
| C538-1-78-2-22-30-2 | 2 | AaBbdd | 41.67 | 39.84 | 15.88 | 3.56 | 6.01 |
| C538-1-78-2-22-30-40 | 2 | AaBbdd | 29.00 | 41.72 | 15.87 | 3.58 | 5.92 |
| C538-1-78-2-22-30-48 | 2 | AaBbdd | 56.00 | 41.90 | 16.18 | 3.61 | 6.03 |
| C538-1-78-2-22-30-57 | 2 | AaBbdd | 36.67 | 43.36 | 16.21 | 3.61 | 5.97 |
| C538-1-78-2-22-30-61 | 2 | AaBbdd | 49.67 | 41.61 | 15.79 | 3.61 | 5.99 |
| C538-1-78-2-22-46-10 | 2 | AAbbdd | 36.67 | 42.09 | 16.27 | 3.59 | 6.04 |
| C538-1-78-2-22-46-12 | 2 | AAbbdd | 40.67 | 40.16 | 15.62 | 3.51 | 6.06 |
| C538-1-78-2-22-46-17 | 2 | AAbbdd | 40.33 | 42.98 | 15.90 | 3.57 | 5.94 |
| C538-1-78-2-22-46-18 | 2 | AAbbdd | 39.67 | 40.67 | 15.56 | 3.53 | 5.94 |
| C538-1-78-2-22-46-19 | 2 | AAbbdd | 49.67 | 39.06 | 15.13 | 3.53 | 5.75 |
| C538-1-78-2-22-46-26 | 2 | AAbbdd | 45.33 | 42.06 | 16.07 | 3.58 | 5.94 |
| C538-1-78-2-22-46-3 | 2 | AAbbdd | 43.00 | 42.25 | 16.19 | 3.63 | 5.98 |
| C538-1-78-2-22-46-5 | 2 | AAbbdd | 53.67 | 40.93 | 15.56 | 3.53 | 5.83 |
| C538-1-78-2-22-46-6 | 2 | AAbbdd | 42.00 | 40.87 | 15.22 | 3.51 | 5.74 |
| C538-1-78-2-22-47-15 | 2 | AAbbdd | 44.67 | 39.03 | 15.23 | 3.52 | 5.77 |
| C538-1-78-2-22-30-19 | 3 | aaBbDD | 50.00 | 41.73 | 15.94 | 3.59 | 5.95 |
| C538-1-78-2-22-30-31 | 3 | aaBbDD | 40.00 | 42.42 | 15.99 | 3.59 | 5.94 |
| C538-1-78-2-22-30-49 | 3 | aaBbDD | 47.33 | 43.31 | 16.35 | 3.67 | 6.00 |
| C538-1-78-2-22-14-10 | 3 | aaBBDd | 47.33 | 39.30 | 15.14 | 3.43 | 5.97 |
| C538-1-78-2-22-14-13 | 3 | aaBBDd | 40.33 | 43.31 | 16.31 | 3.63 | 6.00 |
| C538-1-78-2-22-14-17 | 3 | aaBBDd | 50.00 | 37.93 | 14.97 | 3.48 | 5.79 |
| C538-1-78-2-22-14-18 | 3 | aaBBDd | 47.67 | 39.65 | 15.23 | 3.49 | 6.21 |
| C538-1-78-2-22-14-21 | 3 | aaBBDd | 50.00 | 40.00 | 15.40 | 3.56 | 5.78 |
| C538-1-78-2-22-14-28 | 3 | aaBBDd | 38.67 | 38.28 | 15.19 | 3.45 | 5.90 |
| C538-1-78-2-22-30-35 | 3 | aaBBDd | 45.00 | 42.00 | 15.97 | 3.61 | 5.94 |
| C538-1-78-2-22-30-42 | 3 | aaBBDd | 43.00 | 41.78 | 16.08 | 3.62 | 5.94 |
| C538-1-78-2-22-30-51 | 3 | aaBBDd | 47.00 | 42.34 | 16.12 | 3.62 | 6.02 |
| C538-1-78-2-22-30-52 | 3 | AabbDD | 46.33 | 40.36 | 15.35 | 3.50 | 5.90 |
| C538-1-78-2-22-30-29 | 3 | AaBbDd | 26.67 | 38.50 | 15.27 | 3.51 | 5.78 |
| C538-1-78-2-22-30-58 | 3 | AaBbDd | 42.67 | 40.70 | 15.54 | 3.51 | 5.97 |
| C538-1-78-2-22-30-67 | 3 | AaBbDd | 57.33 | 41.63 | 15.96 | 3.60 | 5.91 |
| C538-1-78-2-22-30-7 | 3 | AaBbDd | 48.00 | 41.32 | 15.85 | 3.54 | 5.99 |
| C538-1-78-2-22-30-69 | 3 | AaBBdd | 31.33 | 39.04 | 15.27 | 3.47 | 5.86 |
| C538-1-78-2-22-30-43 | 3 | AAbbDd | 36.67 | 40.64 | 15.63 | 3.53 | 5.95 |
| C538-1-78-2-22-30-5 | 3 | AAbbDd | 42.67 | 40.39 | 15.57 | 3.54 | 5.87 |
| C538-1-78-2-22-14-16 | 4 | aaBBDD | 48.33 | 40.14 | 15.40 | 3.53 | 5.76 |
| C538-1-78-2-22-14-20 | 4 | aaBBDD | 55.67 | 42.10 | 16.08 | 3.62 | 5.91 |
| C538-1-78-2-22-14-27 | 4 | aaBBDD | 38.33 | 40.52 | 15.78 | 3.54 | 5.90 |
| C538-1-78-2-22-14-9 | 4 | aaBBDD | 35.33 | 35.57 | 14.53 | 3.33 | 5.82 |
| C538-1-78-2-22-30-38 | 4 | aaBBDD | 30.33 | 43.41 | 16.58 | 3.67 | 6.05 |
| C538-1-78-2-22-62-10 | 4 | aaBBDD | 32.00 | 37.50 | 14.84 | 3.39 | 5.89 |
| C538-1-78-2-22-62-11 | 4 | aaBBDD | 37.33 | 33.93 | 14.19 | 3.26 | 5.86 |
| C538-1-78-2-22-62-16 | 4 | aaBBDD | 45.00 | 37.33 | 14.83 | 3.42 | 5.88 |
| C538-1-78-2-22-62-24 | 4 | aaBBDD | 36.00 | 38.89 | 14.91 | 3.47 | 5.76 |
| C538-1-78-2-22-62-37 | 4 | aaBBDD | 37.33 | 40.36 | 15.94 | 3.51 | 6.11 |
| C538-1-78-2-22-62-5 | 4 | aaBBDD | 57.33 | 40.76 | 15.58 | 3.55 | 5.82 |
| C538-1-78-2-22-62-9 | 4 | aaBBDD | 27.33 | 35.85 | 15.04 | 3.39 | 5.96 |
| C538-1-78-2-41-5-1 | 4 | aaBBDD | 37.00 | 41.26 | 15.48 | 3.51 | 5.90 |
| C538-1-78-2-41-5-10 | 4 | aaBBDD | 44.00 | 38.56 | 15.06 | 3.47 | 5.84 |
| C538-1-78-2-41-5-2 | 4 | aaBBDD | 42.67 | 38.91 | 15.11 | 3.47 | 5.84 |
| C538-1-78-2-41-5-3 | 4 | aaBBDD | 53.67 | 32.30 | 13.91 | 3.26 | 5.74 |
| C538-1-78-2-41-5-4 | 4 | aaBBDD | 32.33 | 42.06 | 16.30 | 3.66 | 5.97 |
| C538-1-78-2-41-5-5 | 4 | aaBBDD | 54.33 | 39.20 | 15.40 | 3.51 | 5.85 |
| C538-1-78-2-41-5-6 | 4 | aaBBDD | 30.33 | 39.45 | 15.51 | 3.61 | 5.78 |
| C538-1-78-2-41-5-7 | 4 | aaBBDD | 32.67 | 42.76 | 15.96 | 3.57 | 5.91 |
| C538-1-78-2-41-5-8 | 4 | aaBBDD | 34.00 | 39.02 | 15.43 | 3.50 | 5.83 |
| C538-1-78-2-41-5-9 | 4 | aaBBDD | 40.00 | 39.83 | 15.51 | 3.46 | 5.96 |
| C538-1-78-2-22-30-53 | 4 | AaBbDD | 53.67 | 42.92 | 16.16 | 3.62 | 5.93 |
| C538-1-78-2-22-30-59 | 4 | AaBbDD | 39.00 | 42.05 | 15.96 | 3.58 | 5.93 |
| C538-1-78-2-22-33-3 | 4 | AaBbDD | 33.00 | 37.78 | 15.05 | 3.34 | 5.99 |
| C538-1-78-2-22-62-12 | 4 | AaBbDD | 39.33 | 37.97 | 15.13 | 3.40 | 5.98 |
| C538-1-78-2-22-30-17 | 4 | AaBBDd | 41.67 | 42.40 | 15.86 | 3.58 | 5.91 |
| C538-1-78-2-22-30-4 | 4 | AaBBDd | 35.67 | 38.79 | 14.98 | 3.46 | 5.79 |
| C538-1-78-2-22-30-65 | 4 | AaBBDd | 39.67 | 41.34 | 15.69 | 3.58 | 5.85 |
| C538-1-78-2-22-33-29 | 4 | AAbbDD | 48.33 | 40.76 | 15.63 | 3.54 | 5.86 |
| C538-1-78-2-22-33-33 | 4 | AAbbDD | 38.00 | 40.96 | 15.69 | 3.47 | 5.99 |
| C538-1-78-2-22-33-34 | 4 | AAbbDD | 37.67 | 39.73 | 15.39 | 3.52 | 5.80 |
| C538-1-78-2-22-33-38 | 4 | AAbbDD | 45.33 | 40.81 | 15.81 | 3.55 | 6.03 |
| C538-1-78-2-22-33-6 | 4 | AAbbDD | 36.00 | 33.89 | 14.76 | 3.32 | 5.95 |
| C538-1-78-2-22-33-9 | 4 | AAbbDD | 38.00 | 34.04 | 14.65 | 3.30 | 5.92 |
| C538-1-78-2-22-30-16 | 4 | AABbDd | 38.67 | 39.83 | 15.49 | 3.48 | 5.94 |
| C538-1-78-2-22-30-47 | 4 | AABbDd | 40.00 | 41.17 | 15.80 | 3.56 | 5.97 |
| C538-1-78-2-22-30-6 | 4 | AABbDd | 47.67 | 40.98 | 15.73 | 3.53 | 5.95 |
| C538-1-78-2-22-30-63 | 4 | AABbDd | 54.33 | 43.19 | 16.52 | 3.67 | 6.09 |
| C538-1-78-2-22-30-11 | 4 | AABBdd | 36.33 | 42.66 | 15.70 | 3.58 | 5.85 |
| C538-1-78-2-22-30-64 | 4 | AABBdd | 43.67 | 42.14 | 16.01 | 3.57 | 6.14 |
| C538-1-78-2-22-62-13 | 5 | AaBBDD | 37.33 | 41.52 | 15.81 | 3.55 | 5.97 |
| C538-1-78-2-22-62-14 | 5 | AaBBDD | 42.33 | 38.35 | 15.00 | 3.43 | 5.84 |
| C538-1-78-2-22-62-15 | 5 | AaBBDD | 38.67 | 39.14 | 15.18 | 3.44 | 6.40 |
| C538-1-78-2-22-62-22 | 5 | AaBBDD | 44.33 | 39.25 | 15.17 | 3.47 | 5.86 |
| C538-1-78-2-22-62-25 | 5 | AaBBDD | 40.33 | 41.90 | 15.99 | 3.58 | 5.97 |
| C538-1-78-2-22-62-28 | 5 | AaBBDD | 49.00 | 38.71 | 15.13 | 3.48 | 5.88 |
| C538-1-78-2-22-62-32 | 5 | AaBBDD | 28.33 | 40.12 | 15.35 | 3.53 | 5.80 |
| C538-1-78-2-22-62-34 | 5 | AaBBDD | 40.00 | 39.25 | 15.29 | 3.46 | 5.90 |
| C538-1-78-2-22-62-35 | 5 | AaBBDD | 38.33 | 38.61 | 15.26 | 3.44 | 5.92 |
| C538-1-78-2-22-62-38 | 5 | AaBBDD | 42.33 | 38.50 | 15.18 | 3.45 | 5.93 |
| C538-1-78-2-22-62-40 | 5 | AaBBDD | 38.67 | 40.09 | 15.45 | 3.45 | 5.99 |
| C538-1-78-2-22-28-20 | 5 | AABbDD | 38.00 | 42.63 | 16.21 | 3.59 | 6.08 |
| C538-1-78-2-22-28-6 | 5 | AABbDD | 39.00 | 33.68 | 14.25 | 3.28 | 5.87 |
| C538-1-78-2-22-30-21 | 5 | AABbDD | 54.33 | 40.86 | 15.73 | 3.52 | 5.92 |
| C538-1-78-2-22-30-44 | 5 | AABbDD | 48.67 | 41.51 | 15.67 | 3.52 | 5.93 |
| C538-1-78-2-22-33-12 | 5 | AABbDD | 29.00 | 39.66 | 15.67 | 3.46 | 6.02 |
| C538-1-78-2-22-33-13 | 5 | AABbDD | 51.33 | 41.10 | 15.92 | 3.59 | 5.93 |
| C538-1-78-2-22-33-14 | 5 | AABbDD | 41.00 | 39.59 | 15.51 | 3.49 | 5.91 |
| C538-1-78-2-22-33-18 | 5 | AABbDD | 45.00 | 38.15 | 15.08 | 3.47 | 5.82 |
| C538-1-78-2-22-33-19 | 5 | AABbDD | 59.00 | 32.94 | 14.14 | 3.28 | 5.80 |
| C538-1-78-2-22-33-20 | 5 | AABbDD | 50.67 | 38.36 | 15.30 | 3.52 | 5.77 |
| C538-1-78-2-22-33-26 | 5 | AABbDD | 39.33 | 41.53 | 15.60 | 3.53 | 5.88 |
| C538-1-78-2-22-33-27 | 5 | AABbDD | 47.00 | 40.71 | 15.54 | 3.58 | 5.83 |
| C538-1-78-2-22-33-28 | 5 | AABbDD | 42.00 | 35.95 | 14.81 | 3.41 | 5.80 |
| C538-1-78-2-22-33-32 | 5 | AABbDD | 38.33 | 40.78 | 15.43 | 3.54 | 5.83 |
| C538-1-78-2-22-33-35 | 5 | AABbDD | 38.00 | 41.05 | 15.75 | 3.51 | 5.94 |
| C538-1-78-2-22-33-4 | 5 | AABbDD | 49.33 | 42.23 | 16.26 | 3.60 | 6.00 |
| C538-1-78-2-22-33-5 | 5 | AABbDD | 38.67 | 43.62 | 16.28 | 3.60 | 6.03 |
| C538-1-78-2-22-62-1 | 5 | AABbDD | 34.00 | 39.71 | 15.36 | 3.50 | 5.89 |
| C538-1-78-2-22-62-20 | 5 | AABbDD | 48.00 | 40.56 | 16.42 | 3.60 | 6.07 |
| C538-1-78-2-22-62-29 | 5 | AABbDD | 40.00 | 38.58 | 15.12 | 3.42 | 5.85 |
| C538-1-78-2-22-30-3 | 5 | AABBDd | 41.00 | 39.76 | 15.36 | 3.48 | 5.94 |
| C538-1-78-2-22-11-13 | 6 | AABBDD | 50.33 | 38.61 | 14.70 | 3.47 | 5.60 |
| C538-1-78-2-22-28-1 | 6 | AABBDD | 44.00 | 40.30 | 15.94 | 3.52 | 6.03 |
| C538-1-78-2-22-28-10 | 6 | AABBDD | 42.67 | 37.11 | 14.90 | 3.41 | 5.84 |
| C538-1-78-2-22-28-11 | 6 | AABBDD | 53.00 | 40.31 | 15.87 | 3.52 | 6.03 |
| C538-1-78-2-22-28-12 | 6 | AABBDD | 42.33 | 38.90 | 15.37 | 3.48 | 5.90 |
| C538-1-78-2-22-28-13 | 6 | AABBDD | 47.67 | 37.69 | 14.92 | 3.46 | 5.74 |
| C538-1-78-2-22-28-15 | 6 | AABBDD | 44.00 | 41.36 | 15.59 | 3.56 | 5.78 |
| C538-1-78-2-22-28-16 | 6 | AABBDD | 41.67 | 39.44 | 15.22 | 3.50 | 5.73 |
| C538-1-78-2-22-28-17 | 6 | AABBDD | 28.67 | 38.14 | 14.84 | 3.40 | 5.87 |
| C538-1-78-2-22-28-18 | 6 | AABBDD | 39.00 | 40.85 | 15.67 | 3.46 | 6.02 |
| C538-1-78-2-22-28-2 | 6 | AABBDD | 46.00 | 39.93 | 15.47 | 3.53 | 5.85 |
| C538-1-78-2-22-28-3 | 6 | AABBDD | 39.00 | 41.28 | 15.42 | 3.52 | 5.89 |
| C538-1-78-2-22-28-4 | 6 | AABBDD | 42.00 | 40.95 | 15.75 | 3.58 | 5.90 |
| C538-1-78-2-22-28-7 | 6 | AABBDD | 36.33 | 39.27 | 15.30 | 3.46 | 5.85 |
| C538-1-78-2-22-28-9 | 6 | AABBDD | 39.00 | 38.80 | 15.44 | 3.47 | 5.94 |
| C538-1-78-2-22-33-21 | 6 | AABBDD | 55.00 | 35.27 | 14.46 | 3.33 | 5.77 |
| C538-1-78-2-22-33-23 | 6 | AABBDD | 48.33 | 39.03 | 15.20 | 3.49 | 5.83 |
| C538-1-78-2-22-33-25 | 6 | AABBDD | 42.33 | 40.16 | 15.41 | 3.48 | 5.86 |
| C538-1-78-2-22-33-37 | 6 | AABBDD | 56.67 | 40.88 | 15.88 | 3.55 | 5.98 |
| C538-1-78-2-22-62-19 | 6 | AABBDD | 40.67 | 39.59 | 15.33 | 3.49 | 5.86 |
| C538-1-78-2-22-62-4 | 6 | AABBDD | 40.33 | 39.42 | 15.52 | 3.46 | 5.98 |

**Supplementary Table 6.** Results of RT-PCR analysis performed using isoform-specific primers. The levels of each isoform expression were compared between the wild-type cv. ‘Bobwhite’ (genotype *AABBDD*) and each *TaGS3* mutant (genotypes are shown in column “*TaGS3* genotypes”) using the **2^-∆∆Ct^** method. The levels of *actin* gene expression were used as reference. “A”, “B” and “D” designations are given to the wild-type alleles, and “a”, “b” and “d designations are given to the mutant alleles. The levels of *TaGS3.3* and *TaGS3.5* isoform expression are not detectable in cv. Bobwhite.

| **TaGS3 isoform*** | ***TaGS3* genotype** | **Reps** | **mean(2^-∆∆Ct^)** | **St. err. (2^-∆∆Ct^)** | ***p-value*** | ***p-adjusted* (BH)** |
| --- | --- | --- | --- | --- | --- | --- |
| TaGS3.1 | aabbdd | 5 | 0.497 | 0.230 | 0.099 | 0.173 |
| TaGS3.1 | aaBBDD | 5 | 0.761 | 0.068 | 0.207 | 0.290 |
| TaGS3.1 | AAbbDD | 5 | 0.904 | 0.221 | 0.605 | 0.605 |
| TaGS3.1 | AABBdd | 5 | 0.873 | 0.226 | 0.543 | 0.605 |
| TaGS3.1 | aabbDD | 5 | 0.476 | 0.041 | 0.039 | 0.091 |
| TaGS3.1 | aaBBdd | 5 | 0.318 | 0.027 | 0.019 | 0.065 |
| TaGS3.1 | AAbbdd | 5 | 0.331 | 0.088 | 0.016 | 0.065 |
| TaGS3.2 | aabbdd | 5 | 0.843 | 0.219 | 0.473 | 0.552 |
| TaGS3.2 | aaBBDD | 5 | 0.620 | 0.100 | 0.034 | 0.092 |
| TaGS3.2 | AAbbDD | 5 | 1.067 | 0.280 | 0.921 | 0.921 |
| TaGS3.2 | AABBdd | 5 | 1.553 | 0.166 | 0.039 | 0.092 |
| TaGS3.2 | aabbDD | 5 | 0.708 | 0.066 | 0.061 | 0.106 |
| TaGS3.2 | aaBBdd | 5 | 0.589 | 0.022 | 0.023 | 0.092 |
| TaGS3.2 | AAbbdd | 5 | 3.513 | 2.714 | 0.413 | 0.552 |
| TaGS3.4 | aabbdd | 5 | 0.957 | 0.319 | 0.741 | 0.864 |
| TaGS3.4 | aaBBDD | 5 | 0.444 | 0.103 | 0.047 | 0.160 |
| TaGS3.4 | AAbbDD | 5 | 1.102 | 0.262 | 0.980 | 0.980 |
| TaGS3.4 | AABBdd | 5 | 0.753 | 0.130 | 0.247 | 0.345 |
| TaGS3.4 | aabbDD | 5 | 0.575 | 0.077 | 0.090 | 0.160 |
| TaGS3.4 | aaBBdd | 5 | 0.538 | 0.085 | 0.075 | 0.160 |
| TaGS3.4 | AAbbdd | 5 | 0.580 | 0.072 | 0.092 | 0.160 |

* The levels of *TaGS3.3* and *TaGS3.5* isoform expression are not detectable in cv. ‘Bobwhite’.
